# Pre-Adaptive States and Evolutionary Trajectories in Breast Cancer Drug Resistance: From Drug-Tolerant Persisters to Clonal Evolution

**DOI:** 10.3390/cells15090756

**Published:** 2026-04-23

**Authors:** Hye Young Choi, Mi Jung Park, Seung-Jun Lee, Jeongyun Hwang, Ho-Cheol Choi, Young-Sool Hah

**Affiliations:** 1 Department of Medicine, Gyeongsang National University College of Medicine, 816-15 Jinju-daero, Jinju 52727, Republic of Korea; whitecoc@hanmail.net (H.Y.C.); pichola@naver.com (M.J.P.); 2 Department of Radiology, Gyeongsang National University Hospital, 79 Gangnam-ro, Jinju 52727, Republic of Korea; 3 Institute of Medical Science, Gyeongsang National University College of Medicine, 816-15 Jinju-daero, Jinju 52727, Republic of Korea; 4 Department of Convergence Medical Sciences, Gyeongsang National University, 816-15 Jinju-daero, Jinju 52727, Republic of Korea; 0789zxc@gnu.ac.kr (S.-J.L.); dkdl10252@gnu.ac.kr (J.H.); 5 Biomedical Research Institute, Gyeongsang National University Hospital, 79 Gangnam-ro, Jinju 52727, Republic of Korea

**Keywords:** breast cancer, drug-tolerant persister cells, clonal evolution, epigenetic plasticity, therapy resistance, metabolic reprogramming, tumor microenvironment, pre-adaptive states

## Abstract

**Highlights:**

**What are the main findings?**

- We propose a breast cancer-focused Resistance Continuum as a conceptual framework linking treatment-naïve heterogeneity, pre-adaptive priming, reversible drug-tolerant persister (DTP) states, cycling persisters, and genetically stabilized resistant clones. Rather than implying a universally linear pathway, this framework is intended to organize a canonical resistance trajectory while allowing for subtype-specific and branched evolutionary routes.
- Available evidence across breast cancer subtypes supports an important role for epigenetic and metabolic plasticity in sustaining DTP states. We further discuss epigenetic memory as an emerging, hypothesis-generating concept that may help explain how repeated episodes of persistence could facilitate later resistance in selected contexts.

**What are the implications of the main findings?**

- Mapping candidate intervention points along the continuum highlights opportunities to intercept resistance, including preventing persister emergence, targeting DTP-associated vulnerabilities, and disrupting the transition to stable resistance. However, the strength of supporting evidence is uneven across subtypes and intervention classes, and several strategies remain preclinical or conceptual.
- A technology–stage matrix identifies major evidence gaps in breast cancer, including limited single-cell epigenomic and spatial characterization of cycling persisters and limited tools for directly monitoring non-genetic persistence in patients. In this context, ctDNA is best viewed as a clinically useful monitor of clonal and genetic evolution, whereas direct tracking of non-genetic DTP states remains an important unresolved challenge.

**Abstract:**

Drug resistance is a major cause of treatment failure in breast cancer, yet mutation-centered models do not fully explain delayed resistance, reversible tolerance, or re-sensitization after treatment interruption. Here, we synthesize recent findings in drug-tolerant persister (DTP) biology, clonal evolution, and tumor ecosystem dynamics to propose a breast cancer-focused Resistance Continuum as a conceptual framework for organizing the transition from initial therapy to stable resistance across ER-positive, HER2-positive, and triple-negative disease. Here, we synthesize recent findings in drug-tolerant persister (DTP) biology, clonal evolution, and tumor ecosystem dynamics to propose a breast cancer-focused Resistance Continuum as a conceptual framework for organizing the transition from initial therapy to stable resistance across ER-positive, HER2-positive, and triple-negative disease. This framework describes a canonical, but not universal, trajectory spanning treatment-naïve heterogeneity, pre-adaptive priming, reversible DTP states, cycling persisters, and genetically stabilized resistant clones. We discuss how epigenetic and metabolic plasticity may sustain persistence, and we present epigenetic memory as an emerging hypothesis linking repeated non-genetic persistence to facilitated resistance in selected contexts. We also compare subtype-specific features of DTP biology, outline a multi-omics roadmap for interrogating the continuum, and highlight therapeutic opportunities for resistance interception. Overall, the Resistance Continuum is intended as a working scaffold to integrate current evidence and guide future mechanistic and translational studies.

## 1. Introduction

Breast cancer remains the most frequently diagnosed malignancy among women worldwide, with an estimated 2.3 million new cases and over 660,000 deaths annually according to the latest GLOBOCAN data [1]. In the United States alone, approximately 310,000 women were diagnosed with invasive breast cancer in 2024, underscoring the sustained global burden of this disease [2]. While the advent of molecular subtyping—classifying tumors into estrogen receptor-positive (ER+), human epidermal growth factor receptor 2-positive (HER2+), and triple-negative breast cancer (TNBC) categories—has enabled increasingly precise therapeutic strategies [3,4], treatment failure due to drug resistance continues to account for the majority of breast cancer-related mortality [5]. Indeed, approximately 30% of patients with early-stage breast cancer eventually experience disease recurrence, and drug resistance has been implicated in up to 90% of cancer-related deaths across all malignancies [5,6]. These statistics highlight an urgent and unresolved clinical challenge: despite an expanding arsenal of targeted therapies, endocrine agents, immunotherapies, and antibody–drug conjugates (ADCs), resistance remains the foremost barrier to durable treatment responses in breast cancer [7].

Historically, the mechanisms of drug resistance have been conceptualized through a largely genetic lens. In this classical framework, resistance is viewed as a binary phenomenon: tumor cells are either inherently sensitive or inherently resistant to a given therapy, and the emergence of resistance is attributed primarily to the Darwinian selection of pre-existing genetic variants or the acquisition of de novo mutations under therapeutic pressure [8,9]. Landmark studies have identified well-characterized genetic drivers of resistance in breast cancer, including activating mutations in *ESR1* that confer ligand-independent estrogen receptor activity [10,11], PIK3CA gain-of-function mutations that activate the PI3K/AKT/mTOR signaling axis [12], and *RB1* loss-of-function mutations that enable escape from CDK4/6 inhibitor-mediated cell cycle arrest [13,14]. While these genetic mechanisms are undeniably important and have informed the development of next-generation targeted agents, the mutation-centric model alone fails to account for several key clinical observations—most notably, the phenomenon whereby some patients regain sensitivity to the same therapy after a treatment holiday, a finding that is difficult to reconcile with irreversible genetic alterations [7,15,16].

Over the past decade, a paradigm shift has emerged that reframes drug resistance not as a binary genetic switch but as a dynamic, multi-step evolutionary process [7,17,18]. Central to this revised understanding is the recognition of drug-tolerant persister (DTP) cells—a subpopulation of cancer cells capable of surviving initial therapeutic exposure through reversible, non-genetic adaptations including epigenetic remodeling, transcriptional plasticity, and metabolic reprogramming [19,20,21]. First described in the context of EGFR-mutant lung cancer by Sharma and colleagues in 2010 [21], the DTP phenomenon has since been documented across a wide range of cancer types and treatment modalities, including breast cancer treated with chemotherapy, endocrine therapy, and anti-HER2 agents [22,23,24], melanoma [25,26], and colorectal cancer [27,28]. Critically, accumulating evidence now suggests that the DTP state is not merely a transient curiosity but rather a crucial intermediate stage in a broader resistance trajectory—one that bridges the initial treatment response and the eventual emergence of stably resistant clones through clonal evolution [17,29,30,31]. This trajectory, which we herein term the “Resistance Continuum,” encompasses a series of distinct yet interconnected cellular states: from treatment-naïve heterogeneity, through pre-adaptive epigenetic priming, into the quiescent DTP state, followed by cycling persister expansion, and ultimately culminating in genetically fixed, stably resistant clones.

Despite the rapid expansion of research on DTP biology and clonal evolution, no existing review has systematically integrated these two fields into a unified framework specifically within the context of breast cancer. Prior reviews have made important contributions: Marine et al. catalogued non-genetic resistance mechanisms across cancer types [18]; Vasan et al. provided a comprehensive classification of resistance mechanisms [7]; Pu et al. [20] and Liu et al. [32] reviewed DTP biology in pan-cancer settings; and Laplane and Maley articulated the persistence-to-resistance evolutionary trajectory [17]. However, these frameworks either address resistance at the pan-cancer level without breast cancer subtype stratification or focus on individual components of the resistance process without integrating them into a temporally defined, multi-stage model. Critically, none of these prior frameworks incorporate: (i) a formally staged model with defined temporal dynamics spanning hours to months; (ii) the concept of an epigenetic memory ratchet, whereby residual chromatin modifications from prior DTP episodes progressively lower the barrier to subsequent resistance; (iii) the self-reinforcing epigenetic–metabolic circuit as a unified mechanistic framework sustaining the persister state; (iv) systematic subtype stratification across ER+, HER2+, and TNBC; or (v) a technology–stage matrix mapping current multi-omics capabilities and evidence gaps to each stage of the resistance trajectory. Moreover, the recent explosion of single-cell and spatial multi-omics technologies has generated unprecedented insights into the resistance trajectory at single-cell resolution [33,34], yet these technological advances have not been systematically mapped to each stage of the resistance process in breast cancer.

**Figure 1:**
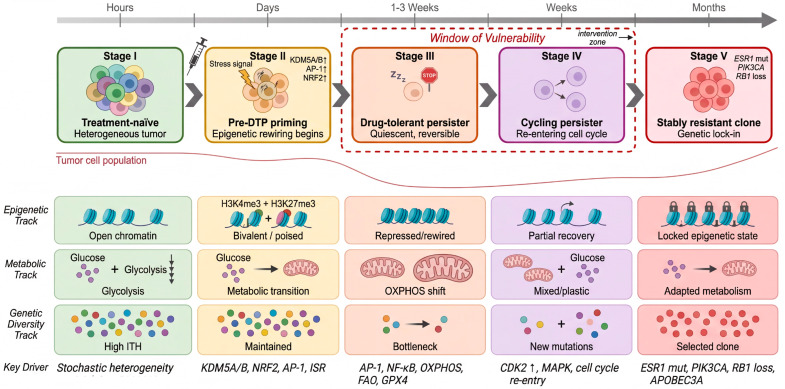
The Resistance Continuum in breast cancer as a conceptual framework. A schematic representation of a canonical resistance trajectory linking five cellular states that may arise during breast cancer treatment: (I) treatment-naïve heterogeneity; (II) pre-DTP priming; (III) reversible drug-tolerant persister (DTP) state; (IV) cycling persister state; and (V) genetically stabilized resistant clone. This model is intended to organize current evidence into a temporally oriented framework and should not be interpreted as implying that all tumors follow a uniform or obligatorily linear sequence. Depending on subtype, therapy class, and tumor ecosystem context, transitions may be branched, overlapping, reversible, or partially bypassed. Four parallel tracks illustrate candidate molecular dimensions associated with each state, including epigenetic remodeling, metabolic adaptation, clonal dynamics, and population behavior. The dashed red rectangle indicates a putative “window of vulnerability” between the emergence of non-genetic persistence and the dominance of stable genetic resistance. The temporal axis spans hours (Stage II) to months (Stage V). Gradient color coding from green (sensitive) through amber, orange, and purple to red (resistant) reflects the progressive acquisition of resistance features. Several transitions—particularly those linking persistence to later resistant states—remain incompletely resolved and should be interpreted as working hypotheses rather than uniformly established pathways. Horizontal arrows indicate the temporal progression from one stage to the next.

In this Review, we present the Resistance Continuum as a breast cancer-focused conceptual framework for organizing the progression from treatment-naïve cellular diversity to stable drug resistance (Figure 1). Our goal is not to imply that all tumors traverse an identical, strictly linear sequence of states, but rather to synthesize evidence supporting a canonical trajectory that may vary in timing, branching structure, and molecular implementation across subtypes and treatment contexts. Furthermore, we provide a technology roadmap for dissecting each stage of the continuum using state-of-the-art multi-omics approaches, and we critically evaluate emerging therapeutic strategies targeting the “window of vulnerability” between DTP emergence and stable resistance. By reframing drug resistance as a continuous evolutionary process rather than a static endpoint [35,36], we aim to provide a conceptual and practical framework for developing next-generation anti-resistance strategies in breast cancer.

## 2. The Resistance Continuum Model: A Conceptual Framework

The emergence of drug resistance in breast cancer is not a singular molecular event but rather a stepwise evolutionary trajectory that unfolds across distinct, temporally ordered cellular states. Drawing on converging evidence from single-cell genomics, epigenetic profiling, and clonal dynamics studies, we present the Resistance Continuum as a conceptual framework comprising five stages (Figure 1). This framework is intended to organize recurrent patterns observed across the literature and should be interpreted as a canonical, but non-universal, trajectory rather than a definitive sequence shared by all tumors.

### 2.1. Defining the Five Stages of the Resistance Continuum

(i) Treatment-naïve state. Prior to any therapeutic intervention, breast tumors harbor substantial intra-tumoral heterogeneity (ITH), encompassing genetically and epigenetically diverse subpopulations [37,38,39,40]. While the bulk of the tumor population is drug-sensitive, a minority of cells exist in transcriptionally or epigenetically “poised” configurations that may predispose them to survive subsequent therapeutic stress—a concept we elaborate upon as pre-adaptive states in Section 3.
(ii) Pre-DTP priming. Upon initial drug exposure, stress-response signaling cascades—including AP-1, NF-κB, and the integrated stress response (ISR)—are rapidly activated within hours to days [22,41,42]. This phase represents the earliest non-genetic adaptation, during which chromatin remodeling enzymes such as *KDM5A*/B begin to redistribute activating histone marks (e.g., H3K4me3) away from lineage-identity genes, while bivalent chromatin states at stress-response loci become resolved toward activation [21,23]. Metabolic rewiring toward mitochondrial oxidative phosphorylation (OXPHOS) also initiates during this window [43,44].
(iii) Drug-tolerant persister (DTP) state. Over the course of days to weeks, a small subpopulation of cells—typically fewer than 5% of the original population—enter a quiescent or slow-cycling state characterized by cell cycle arrest, epithelial-to-mesenchymal plasticity, and markedly altered metabolic dependencies [21,27,45]. This DTP state is fundamentally reversible: upon drug withdrawal, persister cells can re-enter the cell cycle and regenerate a drug-sensitive population phenotypically resembling the original tumor [15,31,46]. Crucially, this reversibility distinguishes DTP cells from classically resistant clones and implies that the tolerance mechanism is primarily epigenetic and transcriptional rather than genetic [47].
(iv) Cycling persister state. A fraction of DTP cells eventually re-enters the cell cycle while maintaining drug tolerance, generating an expanding population of “cycling persisters” [46]. This transition—from quiescent persistence to active proliferation under continued drug pressure—represents a critical juncture in the resistance trajectory, as it greatly expands the population at risk for acquiring stable genetic alterations. Recent lineage-tracing studies have demonstrated that cycling persisters arise from distinct transcriptional programs and represent a phenotypically defined intermediate between fully quiescent DTPs and stably resistant clones [15,46,48].
(v) Stably resistant clone. Over weeks to months, therapy-induced mutagenesis—for example, through *APOBEC3A*-mediated hypermutation [30,49]—and ongoing selection within the cycling persister pool ultimately give rise to clones harboring fixed genetic alterations that confer durable, heritable resistance. In breast cancer, these include canonical resistance drivers such as *ESR1* activating mutations, *RB1* loss, and *PIK3CA* amplification [14,50,51].

To clarify the specific contributions of the present review relative to prior frameworks, Table 1 provides a systematic comparison of the Resistance Continuum Model with four influential existing frameworks [7,17,18,20]. This comparison highlights the five conceptual extensions to prior frameworks offered by the present model: the explicit temporal staging architecture with hours-to-months dynamics, extending the two-phase tolerance-to-resistance trajectory articulated by Laplane and Maley [17]; the epigenetic memory ratchet hypothesis (Section 5.4); the integrated epigenetic–metabolic circuit framework, consolidating observations previously treated separately by Marine et al. [18] and Pu et al. [20]; breast cancer subtype-stratified analysis; and the technology–stage evidence gap matrix.

### 2.2. Genetic Versus Non-Genetic Determinants Across the Continuum

A defining feature of this model is the progressive shift in the dominant mode of adaptation as cells traverse the continuum. The early stages (pre-DTP priming through the quiescent DTP state) are predominantly governed by non-genetic mechanisms—epigenetic remodeling, transcriptional reprogramming, and metabolic plasticity—that are inherently reversible [16,18,36]. As cells transition into cycling persisters, a hybrid regime emerges in which non-genetic plasticity coexists with the stochastic accumulation of genetic alterations [29]. By the final stage, genetic determinism predominates: resistance is locked in by irreversible mutations and is transmitted vertically through cell division. This gradient from non-genetic to genetic control has profound therapeutic implications, as it defines a “window of vulnerability”—the interval between DTP emergence and stable genetic resistance—during which the resistance process may still be reversed or disrupted (Figure 1, dashed box).

### 2.3. Temporal Dynamics: Hours to Months

The five stages of the continuum unfold over markedly different timescales. Pre-DTP priming initiates within hours of drug exposure, with measurable chromatin changes detectable by 24–72 h [21]. The quiescent DTP state typically consolidates over 1–3 weeks of continuous drug exposure in vitro, whereas the transition to cycling persisters occurs over weeks to months in the field [46]. The emergence of stably resistant clones with fixed genetic alterations generally requires months of sustained therapeutic pressure, consistent with clinical observations that acquired resistance to agents such as CDK4/6 inhibitors develops over a median of 12–24 months in metastatic ER+ breast cancer [14,52]. Understanding these temporal dynamics is essential for designing intervention strategies that target the appropriate stage of the continuum.

### 2.4. Conceptual Parallels with Bacterial Persistence

The analogy between cancer DTP cells and bacterial persister cells—first articulated by Sharma et al. [21] and subsequently elaborated by multiple groups [15,53]—provides a conceptual scaffold for the Resistance Continuum Model. In both systems, tolerance precedes resistance, persistence is non-genetic and reversible, and the persister state creates an evolutionary bridge to stable genetic resistance [53]. However, critical differences between the bacterial and cancer systems must be emphasized, as they limit the direct translational value of the analogy. First, cancer DTP cells operate within a complex, spatially organized tissue microenvironment comprising stromal fibroblasts, immune cells, and extracellular matrix components that actively modulate persister cell fate through paracrine signaling, metabolic crosstalk, and immune editing (Section 7)—a layer of regulation entirely absent in bacterial persistence. Second, cancer therapy involves diverse modalities (targeted agents, cytotoxic chemotherapy, immunotherapy, ADCs) that exert qualitatively different selection pressures, whereas bacterial antibiotic resistance typically involves a more limited spectrum of drug mechanisms. Third, cancer cells possess a far more complex genome with extensive epigenetic regulatory capacity, enabling a richer repertoire of non-genetic adaptive states than bacteria. These distinctions underscore that while the bacterial persistence paradigm provides valuable conceptual inspiration, cancer-specific models—such as the Resistance Continuum proposed here—are essential for capturing the full complexity of the persistence-to-resistance trajectory in solid tumors.

## 3. Pre-Adaptive States: The Seeds of Resistance

**Figure 2:**
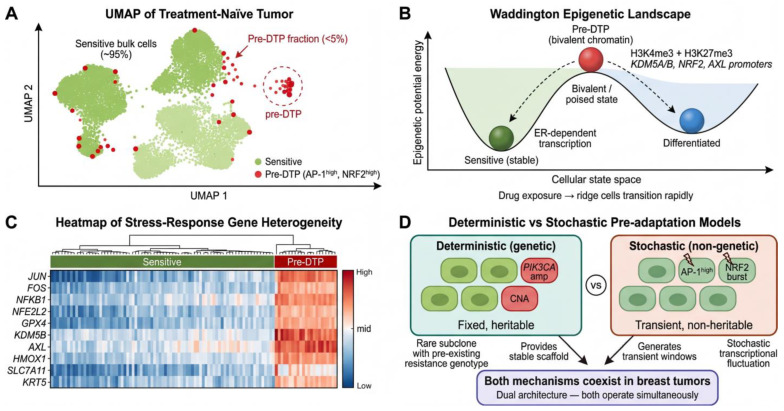
Pre-adaptive states and intra-tumoral heterogeneity in treatment-naïve breast tumors. (**A**) Schematic UMAP representation of a treatment-naïve breast tumor at single-cell resolution. Many cells form multiple sensitive clusters (green), while a rare subpopulation (<5%) of pre-DTP cells (red) is identifiable by elevated expression of stress-response genes (*AP-1*, *NRF2*) and epigenetic regulators (*KDM5B*) prior to any therapeutic exposure. Pre-DTP cells are distributed both as scattered cells at the periphery of sensitive clusters (consistent with stochastic transcriptional fluctuations) and as a distinct mini-cluster (consistent with pre-existing genetic subclones). (**B**) Waddington’s epigenetic landscape metaphor illustrates the concept of chromatin-based pre-adaptation. Drug-sensitive cells occupy deep valleys representing stable transcriptional states, while pre-DTP cells reside on ridges or shallow valleys corresponding to bivalent chromatin configurations (simultaneous H3K4me3 and H3K27me3 marks) at key loci including *KDM5A, NFE2L2* (*NRF2*), and *AXL* promoters. Dashed arrows indicate the potential for rapid state transitions upon drug exposure. (**C**) Heatmap of stress-response and DTP-associated gene expression across single cells in a treatment-naïve tumor. Rows represent 10 key genes (*JUN, FOS, NFKB1, NFE2L2, GPX4, KDM5B, AXL, HMOX1, SLC7A11, KRT5*); columns represent individual cells ordered by hierarchical clustering. A small fraction of cells (right block, red annotation) shows coordinated upregulation of these genes, identifying the transcriptomic signature of the pre-DTP pool. Color scale: blue (low expression) to red (high expression). (**D**) Schematic comparison of deterministic versus stochastic models of pre-adaptation. In the deterministic model (left, teal), specific cells are pre-programmed for survival by fixed genetic features (e.g., pre-existing *PIK3CA* amplification). In the stochastic model (right, coral), survival capacity arises from transient, non-heritable transcriptional fluctuations (e.g., *AP-1* or *NRF2* expression bursts). Current evidence supports a dual architecture in which both mechanisms operate simultaneously in breast tumors (bottom, purple).

A central tenet of the Resistance Continuum Model is that the capacity for drug tolerance does not arise de novo upon treatment but is, to a significant extent, encoded within the heterogeneous landscape of the treatment-naïve tumor. In this section, we examine the evidence that breast tumors contain structured, identifiable pre-adaptive states—genomic, epigenetic, transcriptomic, and metabolic configurations that predispose specific subpopulations to enter the DTP state upon therapeutic challenge (Figure 2).

### 3.1. Evidence for Pre-Existing Resistant Subclones

The question of whether drug resistance arises from the selection of pre-existing variants or from the induction of new alterations under therapy has been a central debate in cancer biology since the pioneering work of Luria and Delbrück in bacterial genetics [54]. In breast cancer, single-cell DNA sequencing studies have provided increasingly direct evidence for the pre-existence model. Kim et al. performed single-cell copy-number profiling of eight TNBC patients treated with neoadjuvant chemotherapy and demonstrated that, in a subset of patients with clonal persistence, a minority of pre-treatment tumor cells already harbored the copy-number profiles observed in post-treatment residual disease [55]. This finding—that rare resistant genotypes were detectable before any therapeutic exposure—strongly supports a model of Darwinian selection acting on pre-existing genetic diversity [56,57]. Earlier single-cell sequencing studies had similarly revealed that aneuploid rearrangements occur early in breast tumor evolution and remain stable during clonal expansion, while point mutations generate extensive subclonal diversity [58,59].

Complementary evidence comes from the PALOMA-3 clinical trial, in which circulating tumor DNA (ctDNA) analysis of 195 patients revealed that multiple resistance-associated subclones, including *ESR1* mutations, were already present at baseline in a substantial fraction of patients randomized to palbociclib plus fulvestrant [14]. More recently, archival single-cell genomics has demonstrated that persistent subclones can be traced back through the pre-invasive stage of breast cancer (DCIS), suggesting that the genetic seeds of resistance may be sown remarkably early in tumor evolution [60]. Breast tumors also maintain a reservoir of subclonal diversity during expansion that can fuel later resistance [61]. Collectively, these studies establish that intra-tumoral genetic heterogeneity (ITH) provides a substrate of pre-existing variants upon which therapeutic selection can act [62], but they also raise a critical question: can pre-adaptation occur in the absence of genetic variation?

### 3.2. Epigenetic Pre-Adaptation: Chromatin States Primed for Survival

Accumulating evidence indicates that epigenetic heterogeneity within treatment-naïve tumors constitutes a non-genetic layer of pre-adaptation that is at least as consequential as genetic ITH. A pivotal mechanism involves bivalent chromatin domains—genomic loci simultaneously marked by the activating modification H3K4me3 and the repressive modification H3K27me3—which maintain genes in a transcriptionally “poised” state, ready for rapid activation or silencing in response to environmental cues [63,64]. In the context of breast cancer, Marsolier et al. demonstrated that pre-existing H3K27me3 patterns at specific loci in TNBC cells conditioned their capacity to tolerate chemotherapy, with cells bearing chromatin configurations showing a markedly higher propensity to enter the DTP state [23].

The histone demethylase *KDM5B* has emerged as a particularly important regulator of epigenetic pre-adaptation in breast cancer. Hinohara et al. showed that *KDM5B* activity drives transcriptomic heterogeneity in ER+ breast cancer cell lines by removing H3K4me3 marks from lineage-identity genes in a stochastic, cell-to-cell variable manner, thereby generating a subpopulation of cells with reduced estrogen-dependent transcriptional programs [65]. These *KDM5B*-high cells exhibited intrinsic resistance to endocrine therapy, and critically, pharmacological inhibition of KDM5 reduced transcriptomic heterogeneity and restored endocrine sensitivity [65]. Similarly, single-cell ChIP-seq analyses have revealed widespread chromatin-state heterogeneity within individual breast tumors, with distinct subpopulations exhibiting differential accessibility at the promoters of stress-response and survival genes [66]. These findings collectively suggest that epigenetic heterogeneity is not merely noise but a structured feature of breast tumors that functionally predisposes specific cells to therapeutic survival.

### 3.3. Transcriptional Heterogeneity as a Bet-Hedging Strategy

Beyond stable chromatin marks, stochastic fluctuations in gene expression—often termed transcriptional “noise”—can generate phenotypic diversity within genetically identical cell populations. This phenomenon has been conceptualized as a biological bet-hedging strategy, analogous to the stochastic phenotype switching observed in bacterial persisters [53,67,68]. In breast cancer, single-cell RNA sequencing of treatment-naïve tumors has revealed that a small fraction of cells constitutively express elevated levels of stress-response transcription factors, including members of the AP-1 family (JUN, FOS), NF-κB pathway components, and interferon-regulatory factor/STAT axis genes, even in the absence of any therapeutic pressure [22,26].

In breast cancer, single-cell and computational studies support the view that pre-treatment transcriptional heterogeneity contributes to later persister emergence. In TNBC, Baudre et al. identified low-level pre-treatment expression of core persister-associated programs, including basal keratins and AP-1/NF-κB/IRF-STAT signaling [23]. In ER+ disease, modeling studies of non-genetic heterogeneity likewise support a bet-hedging interpretation of reversible drug tolerance [69]. Pan-cancer observations, including the melanoma study by Shaffer et al. [27], provide additional conceptual support for this framework.

### 3.4. Metabolic Pre-Conditioning: OXPHOS-High Subpopulations and Redox Homeostasis

Metabolic heterogeneity represents a third dimension of pre-adaptation. Within treatment-naïve breast tumors, a subset of slow-cycling cells exhibits elevated mitochondrial oxidative phosphorylation (OXPHOS) and enhanced antioxidant capacity [44,70,71], suggesting that some cells are metabolically preconditioned for therapeutic survival before drug exposure. Functional support for this interpretation comes from studies showing that persister-like cells are selectively vulnerable to GPX4 inhibition [43] and from evidence that PINK1-mediated mitophagy sustains OXPHOS activity and redox homeostasis in drug-tolerant breast cancer cells [72]. Together, these findings suggest that part of the metabolic infrastructure required for drug tolerance may already be present before therapy begins.

### 3.5. Intra-Tumoral Heterogeneity as the Foundation of the Persister Pool

The preceding subsections describe genetic, epigenetic, transcriptomic, and metabolic dimensions of pre-adaptation separately, but these layers are likely to coexist within the same cells. Although no single study has yet resolved all four dimensions simultaneously in individual breast cancer cells, converging evidence supports a multi-layered architecture in which several pre-adaptive features may compound the probability of therapeutic survival [73]. Emerging multi-modal single-cell platforms such as wellDR-seq [74] may help resolve these integrated pre-adaptive states more directly in future studies.

A key conceptual distinction emerging from this body of evidence is the difference between deterministic and stochastic models of pre-adaptation (Figure 2D). In practice, current evidence suggests that both likely operate together in breast tumors: stable subclonal architecture may provide a scaffold of pre-adaptive risk, whereas stochastic epigenetic and transcriptional fluctuations may create additional transient windows of survival potential. This combined view helps explain why pre-adaptation is best understood as a layered landscape rather than as a single, discrete state.

## 4. Drug-Tolerant Persister Cells in Breast Cancer

**Figure 3:**
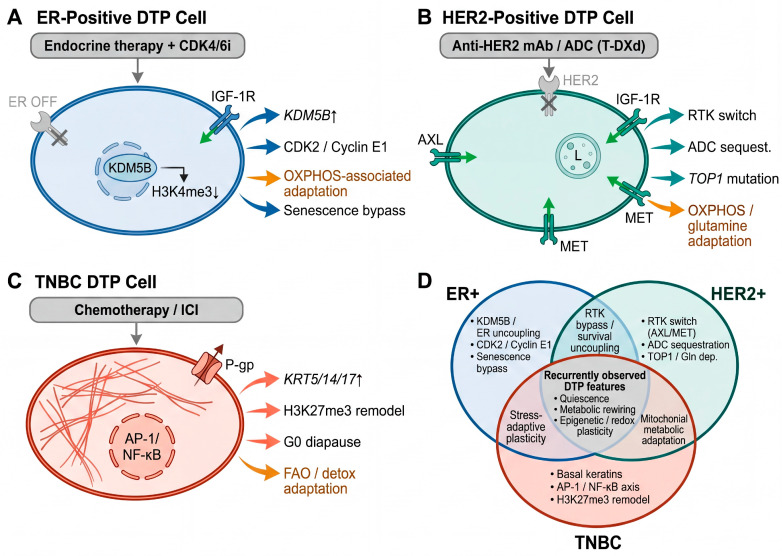
Subtype-specific drug-tolerant persister (DTP) mechanisms across breast cancer subtypes. (**A**) ER-positive DTP cell. Endocrine therapy and CDK4/6 inhibitor exposure promotes a persister-associated state. Labeled arrows summarize representative mechanisms associated with ER+ persistence, including *KDM5B*-mediated H3K4me3 remodeling at estrogen-responsive loci (*KDM5B*↑), CDK2 upregulation and Cyclin E1 amplification enabling bypass of the *RB1*-dependent G1 checkpoint (CDK2/Cyclin E1), OXPHOS-associated metabolic adaptation (amber), and senescence bypass through PI3K/AKT-linked survival signaling. (**B**) HER2-positive DTP cell. Anti-HER2 monoclonal antibodies and antibody–drug conjugates (T-DXd) induce a persister-associated state characterized by receptor tyrosine kinase (RTK) switching to AXL, IGF-1R, and MET (RTK switch), lysosomal ADC sequestration limiting payload delivery (ADC sequest.), payload-specific resistance via TOP1 alterations (TOP1 mutation), and OXPHOS/glutamine-adaptive metabolism (amber). (**C**) Triple-negative breast cancer (TNBC) DTP cell. Chemotherapy or immune checkpoint inhibitor (ICI) exposure promotes a persistence program associated with basal keratin upregulation (KRT5/14/17↑), H3K27me3 remodeling at survival loci (H3K27me3 remodel), diapause-like G0/G1 arrest (G0 diapause), and FAO/detoxification-linked metabolic adaptation (amber). (**D**) Venn diagram synthesizing recurrent versus subtype-enriched DTP features. The central overlap identifies recurrently observed DTP features shared across subtypes, including quiescence, epigenetic/redox plasticity, and metabolic rewiring. Non-overlapping regions indicate subtype-enriched mechanisms, whereas pairwise overlaps highlight partially shared adaptive programs between two subtypes. Metabolic labels are intended to summarize major patterns of adaptive rewiring rather than to imply that any single metabolic phenotype, including OXPHOS dependence, is universal across all breast cancer persister states. Color coding: blue (ER+), teal (HER2+), coral (TNBC), amber (representative metabolic adaptations).

Having established the conceptual framework of the Resistance Continuum and the pre-adaptive landscape that seeds it, we now turn to the central phenotypic state of this trajectory: the drug-tolerant persister (DTP) cell. While the DTP phenomenon has been described across numerous cancer types and treatment modalities, the breast cancer field offers a uniquely informative context due to the well-defined molecular subtypes—ER+, HER2+, and TNBC—each of which employs distinct therapeutic strategies and, as emerging evidence reveals, generates DTP populations with both shared and subtype-specific features (Figure 3).

### 4.1. Defining DTPs: Terminology and Relationship to Related Concepts

Before examining subtype-specific DTP biology, it is essential to clarify the terminological landscape. The terms “drug-tolerant persister,” “dormant cell,” “quiescent cell,” and “cancer stem cell” (CSC) are frequently used in overlapping contexts, generating considerable confusion in the literature [15,75]. In this review, we adopt the operational definition proposed by Russo et al.: DTP cells are cancer cells that survive therapeutic exposure through reversible, predominantly non-genetic mechanisms and can regenerate a drug-sensitive population upon treatment withdrawal [15]. This definition distinguishes DTPs from genetically resistant clones (which harbor irreversible mutations), from dormant/quiescent cells (which may exist in the absence of therapeutic pressure), and from CSCs (which are defined by self-renewal capacity rather than drug tolerance per se), although phenotypic overlap between these categories is well documented [16,20,76]. Hallmark features of the DTP state include: (a) entry into a slow-cycling or G0/G1-arrested state; (b) epigenetic and transcriptional reprogramming; (c) metabolic rewiring, typically toward OXPHOS and fatty acid oxidation; (d) reversibility upon drug removal; and (e) a capacity to serve as an evolutionary reservoir for the emergence of stably resistant clones.

The relationship between DTPs and CSCs warrants deeper examination, as several lines of evidence suggest that these categories may represent overlapping or even identical populations viewed through different experimental lenses. CSCs are operationally defined by their capacity for self-renewal, tumor initiation, and hierarchical differentiation [77], whereas DTPs are defined by drug tolerance and reversibility. However, both populations share key molecular features: slow-cycling/quiescent cell cycle status, elevated expression of drug efflux transporters, activation of developmental signaling pathways (Wnt, Notch, Hedgehog), and metabolic reliance on OXPHOS and fatty acid oxidation [16,20]. Critically, recent single-cell studies have demonstrated that drug exposure can induce the acquisition of CSC-like properties in previously non-stem cells, and conversely, that CSC markers can be transiently expressed in DTP populations [76]. These observations have led some authors to propose that the DTP and CSC phenotypes represent context-dependent manifestations of a shared underlying state of cellular plasticity rather than distinct biological entities [77,78]. In this review, we maintain the DTP-focused nomenclature because it emphasizes the therapeutic context (drug exposure) and reversibility that are central to the Resistance Continuum, while acknowledging that interventions targeting DTP cells may simultaneously affect CSC populations and vice versa.

A related and often-invoked concept is the association between epithelial-to-mesenchymal transition (EMT) and drug tolerance. While EMT-associated transcriptional plasticity is frequently listed among the hallmarks of the DTP state, the relationship between EMT and drug persistence is more nuanced than commonly presented. In breast cancer, partial or hybrid EMT states—in which cells co-express epithelial and mesenchymal markers—have been associated with enhanced drug tolerance and metastatic competence [79,80]. However, full mesenchymal commitment can paradoxically impair drug tolerance in certain experimental contexts, possibly because terminally mesenchymal cells lose the phenotypic plasticity required for reversible state transitions that define the DTP phenotype [81,82]. These findings suggest that the EMT–DTP relationship is context-dependent: partial EMT promotes persistence by expanding the range of accessible phenotypic states, while full EMT may lock cells into a state that is not readily reversible. This distinction carries therapeutic implications, as strategies targeting EMT must consider where along the EMT spectrum a given persister population resides.

To promote terminological clarity throughout the review, we use the following operational definitions. Drug-tolerant persister (DTP) cells are cells that survive initial therapeutic exposure through reversible, predominantly non-genetic mechanisms. Cycling persister cells are a subset of persister-derived cells that have re-entered the cell cycle while retaining drug tolerance under continued therapy. Stably resistant clones are cells that harbor fixed, heritable alterations that confer durable resistance across cell divisions.

### 4.2. DTP States in ER-Positive Breast Cancer

ER+ breast cancer, the most prevalent subtype accounting for approximately 70% of all cases, is treated with endocrine therapy (ET) as the therapeutic backbone, increasingly combined with CDK4/6 inhibitors (CDK4/6i) such as palbociclib, ribociclib, or abemaciclib [83,84]. The DTP state in ER+ breast cancer is characterized by a distinctive set of adaptations that enable survival under estrogen deprivation and cell cycle blockade.

Under endocrine therapy, a subpopulation of ER+ cells survives by transitioning to an ER-independent transcriptional state. Hong et al. performed single-cell transcriptomic profiling of ER+ breast cancer cells exposed to tamoxifen and fulvestrant and identified a multi-step adaptation process in which persister cells progressively downregulated canonical ER-dependent gene expression programs while upregulating alternative survival pathways, including IGF-1R and FGFR signaling [85]. Epigenetically, *KDM5B*-mediated removal of H3K4me3 marks at estrogen-responsive gene promoters plays a central role in this transition, effectively “unlinking” cell survival from estrogen signaling [65]. Of note, this epigenetic rewiring is reversible: upon ET withdrawal, H3K4me3 patterns are restored, and ER-dependent transcription resumes, consistent with the defining reversibility of the DTP state.

Under CDK4/6 inhibitor therapy, an additional layer of tolerance and resistance emerges. Although CDK4/6i are designed to enforce G1 arrest through *RB1*-dependent mechanisms, tumor cells can bypass this blockade through *RB1*-independent cell-cycle rewiring, most notably activation of the cyclin E–CDK2 axis, including CCNE1 overexpression/amplification; complementary genomic analyses have also identified recurrent resistance alterations such as CCNE2 amplification and biallelic *RB1* disruption [86,87,88,89]. Importantly, a substantial proportion of CDK4/6i-tolerant cells do not harbor *RB1* mutations but instead employ non-genetic mechanisms such as senescence bypass and reversible PI3K/AKT pathway activation [13,14,90]. Single-cell transcriptomic analysis of palbociclib-naïve and palbociclib-resistant ER+ cell lines has revealed that transcriptional features of resistance are already detectable in rare cells within the naïve population, with the degree of pre-existing heterogeneity correlating with the palbociclib IC50 [91]. This finding directly connects the pre-adaptive states described in Section 3 to the DTP phenotype in the clinical setting of CDK4/6i-treated ER+ breast cancer. Additionally, CDK4/6 inhibition has been shown to trigger anti-tumor immunity by enhancing antigen presentation, adding another dimension to the persister-immune interaction [92].

### 4.3. DTP States in HER2-Positive Breast Cancer

HER2+ breast cancer provides an important but still less fully resolved setting for the study of drug tolerance within the Resistance Continuum. Compared with ER+ and TNBC disease, where contemporary single-cell transcriptomic and epigenomic studies have more directly characterized persister-associated states, HER2+ DTP biology remains defined primarily by targeted mechanistic studies, bulk profiling, and preclinical models. Accordingly, the HER2+ framework presented here should be interpreted as an emerging working model rather than as a comparably mature map of persister biology.

Under anti-HER2 monoclonal antibody therapy, persister cells frequently exhibit receptor tyrosine kinase (RTK) switching—a phenomenon in which dependence on HER2 signaling is replaced by activation of alternative RTKs, including AXL, IGF-1R, and MET [93,94,95]. This RTK plasticity is largely non-genetic and epigenetically regulated, with chromatin remodeling at RTK gene promoters enabling rapid, reversible shifts in receptor dependency. Metabolically, HER2+ persister cells exhibit a pronounced shift toward OXPHOS and elevated fatty acid oxidation, paralleling the metabolic adaptations observed in ER+ and TNBC DTPs, but with subtype-specific nuances in glutamine metabolism [96].

The emergence of ADCs, particularly T-DXd, has introduced new dimensions to the DTP landscape in HER2+ breast cancer. T-DXd delivers a topoisomerase I inhibitor payload to HER2-expressing cells, but its bystander effect also targets neighboring HER2-low cells. Emerging evidence suggests that ADC tolerance may involve mechanisms distinct from conventional anti-HER2 resistance, including alterations in intracellular drug trafficking, lysosomal sequestration, and modulation of the HER2 antigen presentation density on the cell surface [97]. As ADCs become increasingly central to HER2+ breast cancer treatment, characterizing ADC-specific DTP mechanisms represents an urgent research priority that is only beginning to be addressed.

An additional dimension of ADC tolerance that intersects with DTP biology concerns payload-specific resistance mechanisms. Because ADCs deliver cytotoxic payloads intracellularly upon internalization and lysosomal processing, mutations or changes in expression of the molecular targets of these payloads can confer resistance independently of the mechanisms governing antibody–antigen interactions. For T-DXd specifically, whose payload is the topoisomerase I inhibitor DXd (an exatecan derivative), mutations in TOP1 (topoisomerase I) or upregulation of drug efflux transporters that extrude the free payload from the cytoplasm represent plausible payload-specific resistance mechanisms that have begun to be described in preclinical models [97]. Critically, the relationship between these payload-specific alterations and the DTP state remains unexplored: it is unknown whether DTP cells, by virtue of their slow-cycling status and altered drug metabolism, exhibit differential sensitivity to topoisomerase I-directed payloads compared with actively proliferating cells, or whether the quiescent DTP state provides a pharmacological sanctuary in which payload-mediated DNA damage is reduced simply because replication-dependent toxicity is minimized. Resolving this question has direct clinical relevance, as it would determine whether ADC-tolerant DTP cells represent a reservoir from which payload-resistant clones can subsequently emerge through the acquisition of TOP1 mutations—a trajectory that would extend the Resistance Continuum Model to encompass ADC-specific evolutionary dynamics.

Earlier studies on HER2+ drug tolerance provide important context for the current DTP framework. Zhang et al. demonstrated that lapatinib-tolerant HER2+ breast cancer cells exhibited reversible drug tolerance sustained by epigenetic remodeling rather than genetic mutation, with chromatin landscape changes at RTK gene promoters enabling compensatory signaling through IGF-1R and other alternative receptors [98]. Wilson et al. showed that HER2+ cell lines treated with lapatinib engaged chromatin-mediated transcriptional reprogramming, with upregulation of genes associated with survival signaling and metabolic adaptation, features now recognized as hallmarks of the DTP state [99]. More recently, Hanker et al. provided a comprehensive analysis of resistance mechanisms to anti-HER2 therapies and highlighted the role of HER2 reactivation through structural alterations, as well as the emergence of bypass signaling through PI3K pathway activation, underscoring the interface between initial non-genetic tolerance and eventual genetic resistance in HER2+ breast cancer [100].

Taken together, available data support the view that HER2+ tumors can enter reversible drug-tolerant states characterized by RTK switching, metabolic rewiring, and non-genetic adaptation under anti-HER2 pressure. However, several important features remain insufficiently resolved at single-cell resolution, including the transcriptional heterogeneity of HER2+ DTPs, the chromatin programs that distinguish persisters from later resistant clones, and the degree to which ADC tolerance shares versus diverges from conventional anti-HER2 persistence mechanisms. Proposed ADC-tolerance mechanisms—such as lysosomal sequestration, altered intracellular trafficking, and modulation of HER2 surface density—have thus far been defined largely in bulk systems and preclinical settings. For these reasons, HER2+ persistence should currently be regarded as a high-priority area for longitudinal, patient-linked single-cell and spatial profiling, rather than a fully resolved subtype within the continuum.

### 4.4. DTP States in Triple-Negative Breast Cancer

TNBC, the most aggressive subtype with the fewest targeted treatment options, has paradoxically yielded the most detailed characterization of DTP biology, owing in part to the availability of robust preclinical models and the severe clinical impact of chemoresistance in this context.

Following exposure to standard chemotherapeutic agents (anthracyclines, taxanes, platinum compounds), TNBC cells enter a DTP state characterized by a distinctive transcriptional signature. Baudre et al. recently performed a systematic characterization of persister cells across multiple patient-derived xenograft (PDX) models of TNBC treated with different chemotherapy regimens and identified a shared persistence program that was conserved across both treatment modalities and patients [22]. Hallmarks of this program included upregulation of basal keratins (KRT5, KRT14, KRT17), activation of the stress-response transcription factor networks AP-1, NF-κB, and IRF/STAT, and entry into a diapause-like G0/G1 quiescent state reminiscent of embryonic diapause [22,27,45]. Gene regulatory network analysis identified AP-1, NF-κB, and IRF/STAT as the master transcriptional drivers of the hallmark persister state, suggesting potential therapeutic targets for persister elimination. Single-cell analyses have further revealed that TNBC tumors harbor extensive subclonal heterogeneity that may influence persister emergence [101,102].

Epigenetically, TNBC DTPs are defined by marked H3K27me3 remodeling. Marsolier et al. demonstrated that the pre-existing H3K27me3 landscape at stress-response and survival loci conditioned the capacity of individual TNBC cells to tolerate chemotherapy, with persister cells exhibiting selective derepression of survival genes through loss of the H3K27me3 mark [23]. At the metabolic level, TNBC persisters display the characteristic glycolysis-to-OXPHOS shift and upregulation of lipid metabolism enzymes and P-glycoprotein (P-gp/MDR1)-mediated efflux of toxic lipid peroxidation byproducts, which protects persister cells from ferroptotic cell death [24,42].

With the increasing use of immune checkpoint inhibitors (ICIs) in TNBC [103,104], a nascent but important literature is emerging on DTP states in the context of immunotherapy. Persister cells surviving ICI therapy may employ immune evasion strategies, including downregulation of MHC class I antigen presentation, upregulation of alternative immune checkpoints beyond PD-L1, and remodeling of the local immune microenvironment to exclude cytotoxic T cells [105]. The intersection of DTP biology and immunotherapy resistance in TNBC remains an underexplored but high-priority area for future investigation.

### 4.5. Shared Versus Subtype-Specific DTP Mechanisms: A Comparative Synthesis

Across the three major breast cancer subtypes, several core features of the DTP state are conserved: cell cycle arrest or quiescence, recurrent metabolic rewiring, epigenetic reprogramming involving histone demethylases, upregulation of antioxidant defenses, and transcriptional activation of stress-response programs. For clarity and to avoid repetition across Section 4.2, Section 4.3 and Section 4.4, Table 2 serves as the primary reference for side-by-side comparison of subtype-specific versus recurrent metabolic features. These shared features suggest that the DTP state represents a convergent cellular strategy for surviving diverse therapeutic insults. Pan-cancer parallels provide useful context, but the present discussion is centered on direct evidence from breast cancer whenever such evidence is available.

However, critical subtype-specific differences exist (Table 2). In ER+ breast cancer, the DTP state is closely linked to dissociation of survival from ER-dependent transcription, mediated by *KDM5B* and alternative RTK signaling. In HER2+ breast cancer, RTK switching from HER2 to compensatory receptors emerges as a major adaptive mechanism, although the overall HER2+ persister landscape remains less well resolved than in ER+ and TNBC disease. In TNBC, the persister program is defined more prominently by basal keratin upregulation, the AP-1/NF-κB/IRF-STAT transcriptional axis, and marked stress-adaptive plasticity.

**Table 2:** Comparative features of the drug-tolerant persister (DTP) state across breast cancer subtypes.

Feature	ER+ Breast Cancer	HER2+ Breast Cancer	TNBC	Shared Core
Primary therapy context	Endocrine therapy (tamoxifen, AIs) + CDK4/6 inhibitors	Anti-HER2 mAbs (trastuzumab, pertuzumab), ADCs (T-DXd)	Chemotherapy (anthracyclines, taxanes, platinum); ICI emerging	All induce DTP state through distinct mechanisms
Key epigenetic driver	KDM5B: H3K4me3 removal at ER-responsive promoters	Chromatin remodeling at alternative RTK loci	H3K27me3 remodeling at survival/stress loci (EZH2/PRC2)	KDM5 family activity; global chromatin accessibility changes
Transcriptional reprogramming	ER-independent state; IGF-1R/FGFR bypass signaling	RTK switching: AXL, IGF-1R, MET replace HER2 dependency	AP-1/NF-κB/IRF-STAT axis activation; basal keratins (KRT5/14/17) ↑	Stress-response TF activation; EMT-related plasticity
Cell cycle adaptation	CDK2 upregulation; Cyclin E1 amplification; senescence bypass	Cell cycle plasticity via RTK-downstream signaling	Diapause-like G0/G1 arrest; deep quiescence	Cell cycle arrest/slow-cycling state
Metabolic shift	Glycolysis → OXPHOS; senescence-associated metabolic changes	OXPHOS + glutamine dependency; FAO upregulation	OXPHOS + FAO; P-gp-mediated lipid peroxidation efflux	Glycolysis → OXPHOS switch; FAO activation; NRF2/GPX4 antioxidant defense
Ferroptosis defense	GPX4 upregulation; NRF2 pathway	GPX4/NRF2; membrane lipid remodeling	P-gp (MDR1) efflux of toxic lipid peroxidation byproducts	GPX4 dependency → ferroptosis vulnerability
Drug-specific tolerance	CDK4/6i: RB1-independent bypass (PI3K/AKT); ET: ER uncoupling	ADC: lysosomal sequestration; HER2 antigen density modulation	Multi-drug: P-gp efflux pump upregulation	—
Immune evasion	Limited data; ER+ generally immunologically “cold”	HER2 antigen modulation may reduce ADCC efficacy	MHC-I downregulation; multi-checkpoint upregulation (CD155, galectin-9)	MHC-I loss; PD-L1-independent immune evasion
Reversibility upon drug withdrawal	Yes—ER signaling and H3K4me3 patterns restored	Yes—HER2 dependency may re-emerge	Yes—basal keratin program diminishes; diapause exits	Defining feature of DTP state across all subtypes
Key references	[14,65,85,91]	[97,98,99,100]	[22,23,24,27]	[15,20,21,42]

The rightmost column highlights mechanistic features shared across all three subtypes, representing the conserved DTP core program. ER+, estrogen receptor-positive; HER2+, human epidermal growth factor receptor 2-positive; TNBC, triple-negative breast cancer; OXPHOS, oxidative phosphorylation; FAO, fatty acid oxidation; ADC, antibody–drug conjugate; T-DXd, trastuzumab deruxtecan; AI, aromatase inhibitor; ICI, immune checkpoint inhibitor. ↑ indicates upregulation; → indicates a metabolic shift or transition (e.g., Glycolysis → OXPHOS).

## 5. Epigenetic and Metabolic Plasticity as the Engine of the Continuum

**Figure 4:**
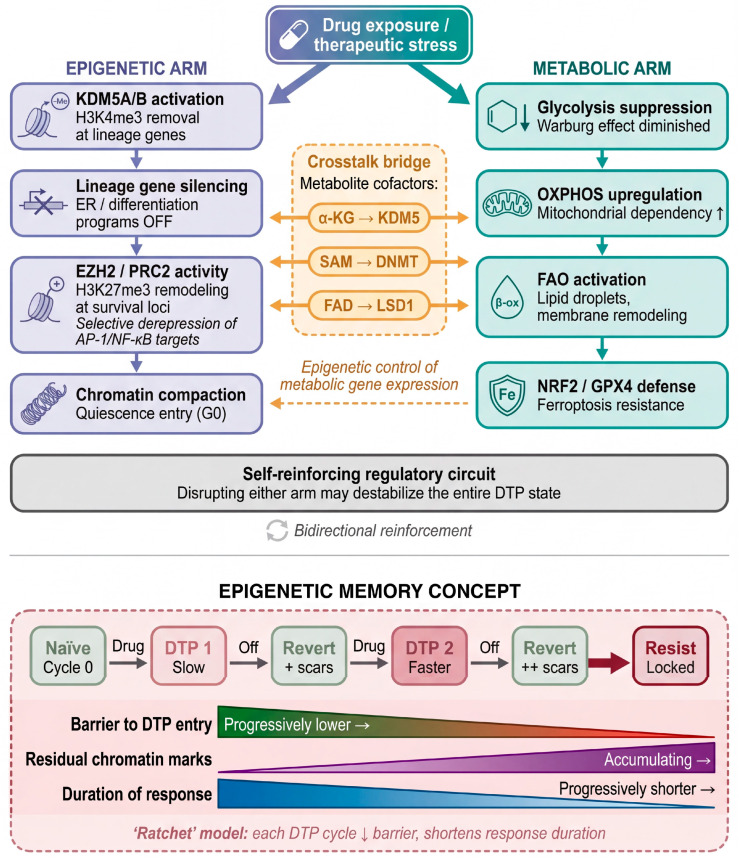
(**Top**) Drug exposure triggers two interconnected adaptive programs: epigenetic remodeling (**left**, indigo), including KDM5A/B-associated H3K4me3 remodeling and chromatin changes linked to DTP formation [21,65,106], and metabolic reprogramming (**right**, teal), including OXPHOS, FAO, and ferroptosis-defense programs reported in persister models [42,43,107,108]. These two arms are hypothesized to be linked through a bidirectional crosstalk bridge in which metabolic intermediates serve as biochemically established cofactors for chromatin-modifying enzymes, while epigenetic modifications are thought to contribute to the expression of metabolic genes. The “self-reinforcing regulatory circuit” framing, therefore, represents a mechanistic inference that requires direct functional validation in breast cancer DTP systems. This schematic is an author-generated integrative summary based on published findings cited in the text and legend, rather than a direct reproduction of any single prior figure. (**Bottom**) Epigenetic memory as an emerging hypothesis: prior passage through the DTP state may leave residual chromatin modifications that incompletely reset after drug withdrawal and could, in selected contexts, facilitate subsequent DTP re-entry or later resistance. The durability, causality, and clinical relevance of these changes remain to be established in longitudinal breast cancer models and patient-linked studies. Arrows in the center bridge indicate bidirectional cofactor exchange between metabolic intermediates and chromatin-modifying enzymes.

Section 3 and Section 4 described the pre-adaptive states that seed the persister pool and the subtype-specific features of the DTP phenotype. In this section, we examine the molecular machinery that sustains the persister state and drives transitions along the Resistance Continuum—focusing on the two deeply interconnected regulatory layers of epigenetic remodeling and metabolic reprogramming (Figure 4). We conclude by introducing epigenetic memory as an emerging, hypothesis-generating concept that may provide a mechanistic bridge between the non-genetic DTP state and the emergence of stably resistant clones.

### 5.1. Epigenetic Remodeling Mechanisms

The DTP state is underpinned by a sweeping reorganization of the chromatin landscape. Three epigenetic modifications have been most extensively implicated: histone demethylation, histone methylation, and changes in chromatin accessibility [63].

Histone demethylases of the KDM5 (JARID1) family occupy a central position in DTP biology. *KDM5A* was identified in the foundational study by Sharma et al. as a critical mediator of the reversible drug-tolerant state in EGFR-mutant lung cancer cells, in which its upregulation drove a global loss of H3K4me3 at lineage-identity gene promoters, effectively silencing differentiation programs and enforcing quiescence [21]. In breast cancer, the paralog *KDM5B* performs an analogous function: its stochastic upregulation in ER+ cells generates transcriptomic heterogeneity by selectively removing H3K4me3 from estrogen-responsive promoters, creating a subpopulation of cells decoupled from ER-dependent survival signaling [65]. Importantly, pharmacological inhibition of KDM5 family enzymes has been shown to collapse this heterogeneity and resensitize persister cells to both endocrine therapy and chemotherapy in preclinical breast cancer models, validating KDM5 as a functionally relevant—and potentially druggable—epigenetic driver of persistence [65,106].

Complementing KDM5-mediated H3K4me3 loss, the Polycomb repressive complex 2 (PRC2) component EZH2, which catalyzes the deposition of the repressive H3K27me3 mark, plays a context-dependent role in persister biology. In TNBC, pre-existing H3K27me3 patterns at specific survival and stress-response loci condition chemotolerance, with selective derepression (H3K27me3 loss) at these loci enabling the activation of the AP-1/NF-κB persister program [23]. The KDM6A/B demethylases that remove H3K27me3 have been implicated in this derepression, and their activity appears to be dynamically regulated during the transition from treatment-naïve to DTP states [23,109]. Furthermore, H4K20me3-mediated repression of inflammatory genes has been identified as a characteristic and targetable vulnerability of persister cells [110], and therapeutically exploitable combinations involving EZH2 inhibition have also been reported in breast cancer, including synthetic lethality with ATM inhibition in BRCA1-deficient models and strong synergy with AKT inhibition in TNBC [111,112].

Beyond individual histone marks, global changes in chromatin accessibility accompany the DTP transition. Single-cell ATAC-seq (scATAC-seq) studies have revealed that persister cells exhibit a distinctive chromatin accessibility profile characterized by the opening of enhancer regions associated with stress-response and developmental gene programs, coupled with the closing of regions linked to proliferative transcription factor binding sites [66,113]. Rosano et al. performed long-term multimodal recording of epigenetic adaptation in dormant breast cancer cells and demonstrated that changes in chromatin accessibility precede transcriptional reprogramming, suggesting that epigenetic remodeling is a cause, not merely a consequence, of the persister transcriptional state [113].

### 5.2. Metabolic Reprogramming in the Resistance Trajectory

In parallel with epigenetic remodeling, DTP cells undergo profound metabolic reprogramming. In breast cancer specifically, Echeverria et al. first demonstrated that chemotherapy-tolerant TNBC cells undergo a marked shift from glycolysis to OXPHOS [43]. We emphasize, however, that the metabolic phenotype of DTP cells is best understood through a dual framework of metabolic dependency and metabolic adaptability, and that OXPHOS dependency—though frequently observed—represents only one of several metabolic strategies that persister cells may employ. This glycolysis-to-OXPHOS switch has been documented in DTP cells across breast cancer subtypes and therapeutic contexts, and appears to serve dual functions: supporting the reduced proliferative rate of persister cells and providing the bioenergetic flexibility required for survival under therapeutic stress [42,43,44,70,114].

Lipid metabolism is a second critical axis of metabolic adaptation. DTP cells upregulate fatty acid oxidation (FAO) pathways, which fuel mitochondrial OXPHOS and generate acetyl-CoA for both energy production and epigenetic modifications (see Section 5.3). Concurrently, persister cells accumulate lipid droplets and remodel their membrane lipid composition, processes linked to protection against oxidative damage and ferroptotic cell death [24,115,116]. In TNBC specifically, P-glycoprotein (P-gp/MDR1)-mediated efflux of toxic lipid peroxidation byproducts provides an additional layer of ferroptosis defense in DTP cells, a mechanism that is reversible upon drug withdrawal as P-gp expression returns to baseline [24].

Redox homeostasis constitutes a third pillar of metabolic adaptation. The shift to OXPHOS inevitably increases mitochondrial reactive oxygen species (ROS) production, necessitating a compensatory upregulation of antioxidant defense systems. DTP cells consistently demonstrate elevated expression of the *NRF2* transcription factor and its downstream targets, including glutathione peroxidase 4 (GPX4), heme oxygenase-1 (HMOX1), and the cystine/glutamate transporter xCT (SLC7A11) [42,117]. The selective vulnerability of DTP cells to GPX4 inhibition, demonstrated by Hangauer et al. across multiple cancer types, including breast cancer models, directly exploits this metabolic dependency and has inspired ongoing efforts to develop ferroptosis-inducing agents as persister-targeting therapies [107,108] (Section 9).

It is important to note, however, that the glycolysis-to-OXPHOS switch, while predominant, is not universal across all DTP contexts. Several studies have identified persister populations that maintain or even increase glycolytic flux under therapeutic stress, particularly in highly glycolytic tumor backgrounds or under specific drug regimens. For example, certain chemotherapy-tolerant breast cancer subpopulations have been shown to upregulate glycolytic enzymes and lactate export rather than transitioning to OXPHOS, suggesting that the metabolic adaptation of DTP cells may be partially dictated by the baseline metabolic wiring of the parental tumor [71,114]. Furthermore, metabolic flexibility—the capacity to switch between glycolysis and OXPHOS depending on nutrient availability and microenvironmental context—may itself be a defining feature of the persister state, rather than a fixed commitment to either metabolic mode [44]. These observations caution against framing OXPHOS dependency as a universal DTP vulnerability and underscore the need to profile patient-specific DTP populations before deploying OXPHOS-targeted therapeutic strategies.

#### Metabolic Dependency Versus Metabolic Adaptability: A Dual Framework

The distinction between metabolic dependency and metabolic adaptability carries direct therapeutic implications. Here, we use metabolic dependency to refer to a state in which DTP survival relies on a pathway that is both essential and relatively non-redundant in a given therapeutic context, whereas metabolic adaptability refers to the capacity of persister cells to switch between fuel sources or energetic programs under changing selective pressures. This distinction matters because pathway inhibition may be effective in dependency-dominant settings but less durable in highly adaptable persister populations [108,118].

Breast cancer subtypes likely differ in the balance between these two dimensions. ER+ persisters may, in selected therapeutic contexts, exhibit greater reliance on OXPHOS-associated programs, particularly under endocrine therapy and CDK4/6 inhibition [43,44,45,72,115]. TNBC persisters may exhibit greater metabolic adaptability, including flexibility across OXPHOS, fatty acid oxidation, and antioxidant defense states [25,43,107]. HER2+ persister metabolism is currently supported by more limited evidence, with available data suggesting OXPHOS and FAO adaptation but with less comprehensive resolution than in ER+ and TNBC settings [97].

### 5.3. Crosstalk Between Epigenetic and Metabolic Programs

A central insight emerging from recent DTP research is that epigenetic and metabolic reprogramming are not independent adaptations but may be linked through bidirectional biochemical crosstalk (Figure 4, center bridge). Metabolites produced by rewired DTP metabolism serve as essential cofactors and substrates for chromatin-modifying enzymes. α-ketoglutarate (α-KG), generated through the tricarboxylic acid (TCA) cycle, is a required co-substrate for the Jumonji-domain histone demethylases, including *KDM5A/B* and KDM6A/B; S-adenosylmethionine (SAM), derived from one-carbon metabolism, is the universal methyl donor for both DNA methyltransferases and histone methyltransferases; and flavin adenine dinucleotide (FAD) serves as a cofactor for the lysine-specific demethylase LSD1/KDM1A [70,119,120]. These relationships are biochemically established, although their precise functional integration in breast cancer DTP systems remains incompletely defined.

Conversely, epigenetic modifications are thought to contribute to the expression of metabolic genes. The opening of chromatin at promoters and enhancers of OXPHOS and FAO genes during the DTP transition may itself be an epigenetically regulated event, plausibly driven by the same histone demethylase and acetyltransferase activities that reshape the broader persister transcriptome [21,65,106]. If experimentally validated, this bidirectional coupling would imply that perturbation of either the epigenetic or metabolic arm of the circuit could destabilize the persister state—a potential principle with therapeutic relevance that is explored further in Section 9. At present, however, the circuit model remains a mechanistic hypothesis rather than a fully established causal framework in breast cancer DTP biology.

The available causal perturbation studies examining this interface in DTP-related contexts remain sparse. In non-breast cancer systems, Roesch et al. demonstrated that blocking mitochondrial respiration in slow-cycling JARID1B-high melanoma cells disrupted the drug-tolerant phenotype [106], supporting the functional importance of metabolic state in persistence. In breast cancer-relevant settings, Luo et al. showed that manipulation of redox signaling altered the equilibrium of breast cancer stem cell states [69], and Fox et al. demonstrated that *NRF2* activation governs redox and nucleotide metabolism during dormant tumor cell recurrence [117]. These studies support the biological plausibility of metabolism-linked state regulation in breast cancer, but they do not directly interrogate epigenetic–metabolic coupling in DTP cells. Thus, current evidence supports a compelling mechanistic model, but not yet a fully validated causal circuit.

### 5.4. Epigenetic Memory: How DTP Experience Accelerates Subsequent Resistance

Perhaps the most consequential feature of the epigenetic dimension of the DTP state is its potential capacity to generate “epigenetic memory.” We note at the outset that the epigenetic memory ratchet concept presented in this section is a hypothesis-generating framework rather than an established feature of breast cancer resistance. Throughout this section, we distinguish between (i) observations directly supported by breast cancer data, (ii) plausible extensions supported by indirect or cross-cancer evidence, and (iii) predictions of the ratchet model that remain untested. Although the DTP state is operationally defined by its reversibility—persister cells can re-enter the cell cycle and regenerate drug-sensitive populations upon treatment withdrawal—emerging evidence suggests that this reversal may be incomplete. In this section, we first review the direct experimental evidence for residual chromatin marks following DTP reversion, then articulate a ratchet hypothesis linking these observations to the progressive acceleration of resistance, and finally propose experimental designs to test this model.

#### 5.4.1. Direct Evidence for Residual Chromatin Marks After DTP Reversion

The most direct evidence for incomplete epigenetic reversal in breast cancer comes from Rosano et al., who performed long-term multimodal epigenetic recording in dormant breast cancer cells. Using parallel profiling of DNA methylation and histone modifications, they demonstrated that cells emerging from a prolonged quiescent state retained altered DNA methylation patterns and histone modification profiles at key stress-response and survival loci, even after weeks of drug-free culture and apparent phenotypic reversion to a proliferative state [113]. Critically, these residual marks were not randomly distributed but were concentrated at genomic loci functionally linked to stress tolerance and survival signaling, suggesting a structured rather than stochastic retention process.

Complementary findings from other cancer types support the generality of this phenomenon. In EGFR-mutant lung cancer, He et al. reported that DTP cells that reverted to a drug-sensitive state upon ALK inhibitor withdrawal exhibited heritable epigenetic changes, including persistent chromatin marks associated with IRS1 phosphorylation signaling, that correlated with increased frequency of persister re-emergence upon drug rechallenge [119]. Russo et al. similarly reported that the efficiency of persister-state re-entry increased with prior DTP experience in colorectal cancer models [15]. While these cross-cancer observations are consistent with a general principle of epigenetic mark retention, it is important to note that direct demonstration of retained chromatin modifications specifically following drug-induced (rather than dormancy-associated) DTP reversion in breast cancer cell lines or patient specimens has not yet been reported, representing a critical evidence gap.

#### 5.4.2. The Ratchet Hypothesis: Progressive Lowering of Re-Entry Barriers

Building on the evidence for residual chromatin marks described above, we propose the “epigenetic memory ratchet” hypothesis. We emphasize, however, that this model rests on several important evidentiary limitations: current support is largely based on single-episode observations, much of the literature is indirect or cross-cancer, and causal links between retained chromatin marks and accelerated DTP re-entry remain to be demonstrated. With these limitations stated explicitly, the ratchet framework can be used to generate testable predictions about how prior DTP experience might influence future resistance trajectories. In this model, the first DTP episode leaves a modest residual mark at stress-response and survival loci; the second episode, initiating from a partially pre-primed chromatin state, proceeds more rapidly and deposits additional modifications; and each successive cycle further ratchets the system toward a chromatin configuration that facilitates increasingly efficient DTP re-entry—ultimately accelerating the trajectory from reversible tolerance to irreversible genetic resistance.

If validated, this hypothesis would have significant implications for the Resistance Continuum Model. It would predict that the “window of vulnerability” between DTP emergence and stable genetic resistance narrows with each successive treatment cycle, potentially explaining the common clinical observation that successive lines of therapy in metastatic breast cancer yield progressively shorter durations of response [121]. It would also imply that strategies aimed at erasing or preventing the accumulation of epigenetic memory—for instance, through early-line combination of standard therapy with epigenetic modifiers—could slow the pace of resistance evolution, a concept we examine in Section 9. However, we emphasize that the ratchet model currently remains a hypothesis extrapolated from single-episode observations of chromatin mark retention; the critical prediction of progressive, cycle-dependent accumulation has not yet been directly tested in any cancer type. Importantly, all supporting evidence to date derives from in vitro cell line models and preclinical systems. No clinical study has yet profiled the chromatin landscape of patient tumors across multiple sequential treatment cycles to determine whether residual epigenetic marks genuinely accumulate in human breast cancers with each successive line of therapy. The commonly observed clinical pattern of progressively shorter response durations across successive treatment lines in metastatic breast cancer is consistent with the ratchet hypothesis but is also explainable by alternative mechanisms, including the accumulation of genetic resistance mutations and the progressive depletion of effective therapeutic options. Establishing the ratchet model in human tumors will require longitudinal epigenomic profiling of matched patient specimens collected at multiple treatment-relapse cycles—a technically challenging but now feasible endeavor with the advent of single-cell CUT&Tag and related technologies (Section 8).

Several key pieces of evidence remain lacking. First, no study has performed serial drug challenge experiments in breast cancer models with epigenomic profiling at each intervening withdrawal phase to quantify the cumulative accumulation of residual marks. Second, the relationship between the number of prior DTP episodes and the kinetics of persister re-entry (the central prediction of the ratchet model) has not been systematically measured. Third, it remains unclear whether the retained chromatin modifications are truly cumulative or whether they reach a plateau after one or two DTP cycles. Fourth, the causal relationship between residual marks and accelerated re-entry—as opposed to a merely correlative association—has not been established through gain- or loss-of-function experiments targeting specific chromatin modifiers at specific loci.

#### 5.4.3. Experimental Designs to Test the Ratchet Model

We propose three complementary experimental approaches to rigorously test the epigenetic memory ratchet hypothesis in breast cancer. First, a serial drug challenge protocol with intervening epigenomic profiling: breast cancer cell lines (ER+, HER2+, and TNBC) would be subjected to three or more successive cycles of drug treatment followed by drug withdrawal until phenotypic reversion, with single-cell CUT&Tag (scCUT&Tag) profiling of H3K4me3, H3K27me3, and H3K27ac performed at the end of each withdrawal phase. This would directly test whether residual histone marks accumulate progressively across cycles and identify the specific genomic loci at which memory is deposited.

Second, quantification of DTP re-entry kinetics across successive cycles: using DNA barcoding to track individual clonal lineages, the time to DTP state entry, the fraction of cells entering the persister state, and the time to phenotypic reversion would be measured across each successive drug challenge cycle. A progressive shortening of DTP entry time and/or an increase in persister fraction across cycles would provide functional evidence for the ratchet mechanism, while barcoding would distinguish between the selection of pre-adapted lineages and genuine epigenetic memory within individual lineages.

Third, causal validation through targeted epigenetic editing: CRISPR-based epigenome editing tools (e.g., dCas9-DNMT3A for targeted DNA methylation, dCas9-KRAB for targeted H3K9me3 deposition) would be deployed to either erase residual marks at identified memory loci after DTP reversion or to artificially install such marks in treatment-naïve cells. If erasing residual marks eliminates the accelerated re-entry phenotype, or if installing marks in naïve cells recapitulates it, this would establish the causal role of specific chromatin modifications in the ratchet mechanism.

Taken together, the epigenetic memory ratchet should currently be used to generate mechanistic and translational hypotheses for future study rather than to inform near-term clinical decision-making.

## 6. From Persisters to Resistant Clones: Clonal Evolution Under Therapy

**Figure 5:**
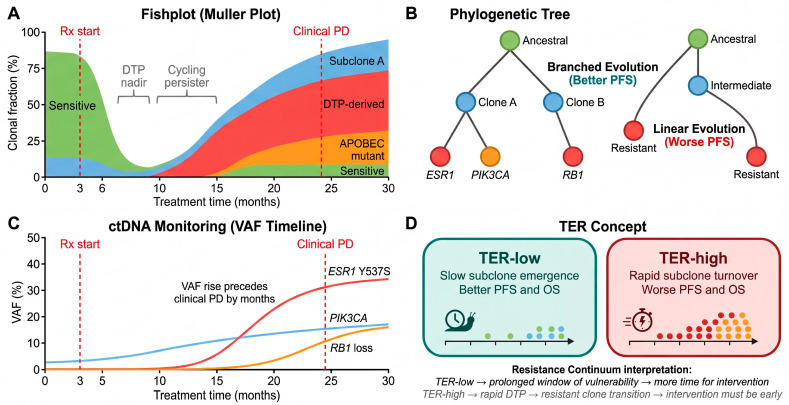
Clonal evolution dynamics from drug-tolerant persisters to resistant clones. (**A**) Fishplot (Muller plot) depicting the temporal evolution of clonal composition under therapy. Treatment initiation (red dashed line) triggers massive elimination of the sensitive population (green), followed by a bottleneck at the DTP nadir. DTP-derived clones (red) and *APOBEC3A*-driven mutant subclones (amber) emerge during the cycling persister phase and progressively expand, ultimately dominating the resistant population. (**B**) Schematic phylogenetic trees illustrating two evolutionary patterns observed in metastatic breast cancer: branched evolution (top), associated with greater clonal diversity and slower disease progression, and linear evolution (bottom), associated with rapid sequential selection and worse prognosis. (**C**) Circulating tumor DNA (ctDNA) monitoring of variant allele frequencies (VAF) for resistance-associated mutations. Rising VAF for ESR1 Y537S and *PIK3CA* mutations precedes clinical disease progression (Clinical PD, red dashed line) by several months, providing a potential real-time window for therapeutic intervention during the cycling persister phase. (**D**) The tumor clonal evolution rate (TER) concept: TER-low tumors exhibit slow subclone emergence and a prolonged window of vulnerability amenable to intervention, whereas TER-high tumors undergo rapid clonal turnover requiring early, aggressive anti-resistance strategies. Clone colors are consistent across all four panels to enable cross-referencing.

The preceding sections established that the DTP state is sustained by reversible, non-genetic mechanisms—epigenetic remodeling, metabolic rewiring, and transcriptional plasticity. Yet the clinical reality of drug resistance in breast cancer is ultimately defined by the emergence of irreversible, genetically fixed resistant clones. How does the system transition from non-genetic tolerance to genetic resistance? In this section, we examine the final stages of the Resistance Continuum: the DTP-to-cycling persister transition, therapy-induced mutagenesis within the persister pool, and the clonal selection dynamics that give rise to stably resistant populations (Figure 5).

### 6.1. The DTP-to-Cycling Persister Transition: Molecular Gatekeepers

The transition from quiescent DTP cells to actively cycling persisters represents the critical inflection point at which reversible tolerance acquires the proliferative capacity to fuel clonal expansion. Oren et al. demonstrated, using DNA barcoding and single-cell RNA sequencing in EGFR-mutant lung cancer models, that cycling persisters arise from specific lineages within the DTP population that activate distinct transcriptional programs, including upregulation of antioxidant metabolism and reactivation of proliferative signaling through MAPK and mTOR pathways [46]. Although equivalent lineage-tracing resolution has yet to be achieved in breast cancer models, converging evidence suggests that analogous gating mechanisms operate. In ER+ breast cancer, the re-entry of CDK4/6 inhibitor-tolerant cells into the cell cycle has been linked to CDK2 upregulation and cyclin E1 amplification, which bypasses the *RB1*-dependent G1 checkpoint enforced by CDK4/6 inhibitors [14,86,88]. In TNBC, the exit from diapause-like quiescence appears to involve reactivation of MYC-driven transcription and re-engagement of receptor tyrosine kinase signaling [27,43]. Crucially, this transition expands the population at risk for acquiring de novo genetic alterations, as actively dividing cells are inherently more susceptible to replication errors and mutagenic processes [122].

### 6.2. Mutagenesis Within the DTP State: Therapy-Induced Versus Pre-Existing Variants

APOBEC-signature mutagenesis is especially relevant to breast cancer, where it represents one of the most prevalent mutational processes and has been implicated in clonal diversification and therapeutic resistance across multiple treatment contexts [123,124]. In this disease-specific context, a landmark discovery linking DTP biology to clonal evolution was the identification of therapy-induced mutagenesis as an active process within persister cells. Isozaki et al. demonstrated that drug-tolerant persister cells in multiple cancer types, including breast cancer models, upregulate the *APOBEC3A* cytidine deaminase, generating a burst of C-to-T and C-to-G mutations at APOBEC signature motifs (TCW context) during the DTP state [30]. This therapy-induced *APOBEC3A* activity provides a direct mechanistic link between the non-genetic DTP state and the generation of genetic diversity from which resistant clones can be selected. Furthermore, blocking genomic instability has been shown to prevent acquired resistance to MAPK inhibitor therapy in melanoma, suggesting that related anti-mutagenic strategies may also warrant investigation in breast cancer [49].

This therapy-induced mutagenesis operates in concert with, rather than as a replacement for, the selection of pre-existing genetic variants described in Section 3. The relative contribution of each mechanism is likely context-dependent: in tumors with high baseline ITH, pre-existing resistant subclones may dominate the post-treatment landscape, whereas in tumors with lower initial genetic diversity, therapy-induced mutagenesis within the DTP pool may be the primary source of resistance-conferring mutations [125,126]. Distinguishing these contributions in individual patients remains a major analytical challenge, but one that emerging technologies such as longitudinal ctDNA sequencing and single-cell multi-omics are beginning to address (Section 8).

### 6.3. Branched Versus Linear Evolution in Breast Cancer

The pattern of clonal evolution under therapy is not uniform across breast cancer patients. Lv et al. performed systematic clonal analysis of metastatic breast cancer patients using next-generation sequencing with PyClone and CITUP computational frameworks and identified two predominant evolutionary patterns: branched evolution, in which multiple subclones diverge from the ancestral lineage and coexist, generating extensive clonal diversity; and linear evolution, in which a single dominant clone sequentially acquires resistance-conferring alterations [50]. Strikingly, patients with branched evolution—characterized by greater clonal diversity—demonstrated slower disease progression than those with linear evolution [50]. This finding, while initially unexpected, is consistent with predictions from evolutionary ecology—specifically, the competitive exclusion principle, which posits that high clonal diversity imposes competitive constraints that slow the dominance of any single clone—whereas linear evolution represents unimpeded sequential selection toward a fitness optimum [57,62,127].

In the context of the Resistance Continuum, branched evolution likely reflects the diversity of DTP states and cycling persister lineages coexisting within a single tumor, each exploring different adaptive trajectories. Linear evolution, by contrast, may indicate that a single pre-adaptive state or DTP lineage has a decisive fitness advantage, rapidly channeling the evolutionary trajectory toward a specific resistance genotype. Distinguishing these patterns prospectively could inform therapeutic strategy: branched evolution may be more amenable to adaptive therapy approaches that exploit inter-clonal competition, whereas linear evolution may require early, aggressive intervention to pre-empt the dominant resistance trajectory [121,128].

### 6.4. The Tumor Clonal Evolution Rate as a Prognostic Indicator

Recognizing that the speed of clonal evolution, not merely its pattern, carries prognostic significance, Lv et al. introduced the concept of the tumor clonal evolution rate (TER)—a quantitative metric reflecting the pace at which new subclones emerge under therapeutic pressure [50]. In their multicenter study of 406 metastatic breast cancer patients, patients in the TER-low group demonstrated significantly better progression-free and overall survival than those in the TER-high group, independent of conventional clinicopathological prognostic factors [50]. The TER concept aligns naturally with the Resistance Continuum Model: a high TER may reflect rapid traversal through the DTP → cycling persister → resistant clone trajectory, potentially driven by strong pre-adaptive states, aggressive therapy-induced mutagenesis, or a permissive tumor microenvironment (Section 7). Conversely, a low TER may indicate that the evolutionary process is stalled at the DTP or cycling persister stage, representing a prolonged window of vulnerability during which the resistance trajectory might still be disrupted.

### 6.5. Liquid Biopsy and ctDNA as Clinical Monitors of Clonal and Genetic Evolution

The translational value of the Resistance Continuum depends in part on the ability to monitor resistance dynamics during therapy. In current clinical practice, circulating tumor DNA (ctDNA) is the most accessible tool for tracking clonal and genetic evolution in breast cancer, particularly the emergence and expansion of resistance-associated mutations under treatment pressure [129,130]. In the PALOMA-3 trial, serial ctDNA profiling revealed that clonal evolution occurs frequently during CDK4/6 inhibitor therapy, with new driver mutations in *PIK3CA* and *ESR1* emerging in both treatment arms, while *RB1* mutations appeared selectively in the palbociclib-treated arm [14]. Notably, the detection of increasing variant allele frequencies (VAFs) for resistance-associated mutations preceded clinical progression by weeks to months, raising the possibility of intervention during the cycling persister stage—before fully resistant clones have achieved dominance [131].

At the same time, the interpretive scope of ctDNA should not be overstated. ctDNA is well-suited to monitoring genetically encoded resistance trajectories, but it does not directly capture non-genetic drug-tolerant persister (DTP) states, especially during early persistence phases, when chromatin remodeling, quiescence, and metabolic rewiring predominate in the absence of stable genomic alterations. Thus, within the Resistance Continuum, ctDNA is best positioned to inform the later transition from persistence toward clonal outgrowth rather than to serve as a direct biomarker of the DTP state itself.

Emerging clinical trial designs are beginning to exploit this principle. The PADA-1 trial demonstrated that switching to fulvestrant and palbociclib upon detection of rising *ESR1* mutations during aromatase inhibitor and palbociclib therapy significantly improved progression-free survival [132]. The plasmaMATCH trial further validated the use of ctDNA-directed therapy selection in advanced breast cancer [133]. The SAFIR 03–ARRIBA trial is prospectively evaluating whether early ctDNA-guided therapy switching in patients with rising PIK3CA ctDNA levels during CDK4/6 inhibitor therapy can delay clinical progression, effectively translating the Resistance Continuum concept into an actionable clinical strategy. More broadly, integrating ctDNA dynamics with computational models of clonal evolution holds promise for predicting the trajectory of resistance in individual patients and tailoring the timing and selection of subsequent therapies accordingly [134]—a vision we discuss further in Section 8 and Section 9.

## 7. Tumor Microenvironment Interactions Along the Resistance Continuum

The preceding sections have focused primarily on cell-autonomous mechanisms that drive progression along the Resistance Continuum—epigenetic remodeling, metabolic reprogramming, and clonal evolution within the tumor cell population itself. However, breast tumors are complex ecosystems in which cancer cells continuously interact with stromal fibroblasts, immune cells, endothelial cells, and the extracellular matrix [35,135]. Accumulating evidence indicates that the tumor microenvironment (TME) is not merely a passive bystander in the resistance trajectory but an active participant that shapes persister cell fate at multiple stages of the continuum [136]. In this section, we examine the TME as a contextual modulator of DTP biology, focusing on three principal axes: cancer-associated fibroblast (CAF)-mediated niche protection, immune cell interactions, and hypoxia-driven metabolic crosstalk.

### 7.1. Cancer-Associated Fibroblast-Mediated Niche Protection of DTP Cells

Cancer-associated fibroblasts (CAFs) constitute the most abundant stromal cell type in the breast cancer TME and have been increasingly recognized as active contributors to therapy resistance [137,138]. Several lines of evidence suggest that CAFs provide a protective niche that promotes DTP cell survival. Paracrine signaling through secreted factors—including IL-6, HGF, and members of the FGF family—can activate survival pathways (JAK/STAT3, PI3K/AKT, MAPK) in neighboring tumor cells, mimicking or reinforcing the signaling programs that characterize the DTP state [139,140]. In ER+ breast cancer specifically, CAF-derived growth factors have been shown to sustain ER-independent survival signaling in tumor cells under endocrine therapy, effectively lowering the barrier to DTP entry [141,142].

Beyond soluble factors, CAFs contribute to niche protection by remodeling the extracellular matrix (ECM). Increased ECM stiffness and collagen deposition around residual tumor cells can impede drug penetration, creating pharmacological sanctuaries that promote local drug tolerance independent of cell-intrinsic adaptation [143]. Furthermore, recent single-cell and spatial transcriptomics studies have revealed that CAF populations are themselves heterogeneous, with functionally distinct CAF subtypes—including inflammatory CAFs (iCAFs) and myofibroblastic CAFs (myCAFs)—that are preferentially enriched in different spatial compartments of the tumor [144,145,146]. The spatial co-localization of specific CAF subtypes with residual tumor cells after therapy suggests that the persister niche is not randomly distributed but is organized into defined microanatomical compartments, a hypothesis that spatial omics technologies are now beginning to test directly (Section 8).

### 7.2. Immune Cell Interactions: Macrophage Polarization and T-Cell Exclusion

The immune microenvironment undergoes significant remodeling during the DTP transition, with consequences for both persister cell survival and the efficacy of immunotherapy [147]. Tumor-associated macrophages (TAMs), the most abundant immune cell type in breast tumors, exhibit remarkable phenotypic plasticity. Under therapeutic stress, the macrophage population shifts toward an immunosuppressive, M2-like polarization state, characterized by the expression of anti-inflammatory cytokines (IL-10, TGF-β) and tissue remodeling factors that collectively support tumor cell survival and immune evasion [148,149]. Lipid-associated macrophages (LAMs), a recently identified TAM subset enriched in breast cancer, have been spatially mapped to the tumor–stroma interface and are associated with immunosuppressive signaling and poor prognosis [146].

DTP cells themselves may actively shape the immune microenvironment to promote their own survival. Downregulation of MHC class I molecules and the antigen-processing machinery has been documented in persister cells across multiple cancer types, reducing their visibility to cytotoxic CD8+ T cells [105]. Concurrently, DTP cells can upregulate ligands for inhibitory receptors beyond the classical PD-1/PD-L1 axis, including CD155 (TIGIT ligand), galectin-9 (TIM-3 ligand), and HLA-G, creating a multi-layered immune checkpoint barrier that may explain the limited efficacy of single-agent PD-1/PD-L1 blockade in most breast cancer subtypes [22,105]. In the context of the Resistance Continuum, this immune remodeling represents a co-evolutionary process: as tumor cells traverse the DTP state, the immune microenvironment is simultaneously reshaped to tolerate their presence, creating a self-reinforcing niche of immune privilege.

### 7.3. Hypoxia and Metabolic Crosstalk Between Stroma and DTP Cells

Intratumoral hypoxia, a pervasive feature of the breast cancer TME, intersects with DTP biology through multiple mechanisms. Hypoxia-inducible factor 1α (HIF-1α) activation drives transcriptional programs that overlap substantially with the DTP gene expression signature, including upregulation of glycolytic enzymes, angiogenic factors, and epithelial-to-mesenchymal transition (EMT) markers [150]. Hypoxic niches within the tumor may therefore serve as natural incubators for the persister phenotype, even prior to therapeutic intervention—a possibility that links TME architecture to the pre-adaptive states described in Section 3. This spatial organization of the TME has been further illuminated by spatial genomics approaches that map clonal structure onto tissue architecture [151,152].

Metabolic crosstalk between stromal and tumor cells adds further complexity. CAFs and TAMs can supply DTP cells with metabolic intermediates—including lactate, glutamine, and fatty acids—that fuel the OXPHOS and FAO programs sustaining the persister state [153]. This metabolic symbiosis creates a dependency that may represent a therapeutic vulnerability: disrupting stromal metabolite supply could starve DTP cells of the substrates required to maintain their adapted metabolic program. However, targeting these interactions therapeutically is complicated by the bidirectional nature of the crosstalk and the risk of off-target effects on normal tissue metabolism.

### 7.4. The Persister Niche as a Spatially Defined Microenvironmental Compartment

A unifying theme across the CAF, immune, and hypoxia axes is that the persister-supportive microenvironment is not uniformly distributed but is spatially organized into discrete niches within the tumor. Spatial transcriptomics studies of breast tumors treated with neoadjuvant therapy have begun to reveal that residual tumor cells after treatment cluster in defined microanatomical locations characterized by high CAF density, M2 macrophage enrichment, hypoxic gene signatures, and reduced CD8+ T-cell infiltration [154,155]. These “persister niches” may represent spatially resolved versions of the protective microenvironment described above, and their identification through spatial profiling could provide biomarkers for predicting and monitoring the DTP state in clinical specimens. The integration of spatial omics with the technological approaches described in Section 8 represents one of the most promising frontiers for translating TME biology into actionable resistance monitoring strategies.

## 8. Technology Roadmap: Dissecting the Continuum with Multi-Omics

**Figure 6:**
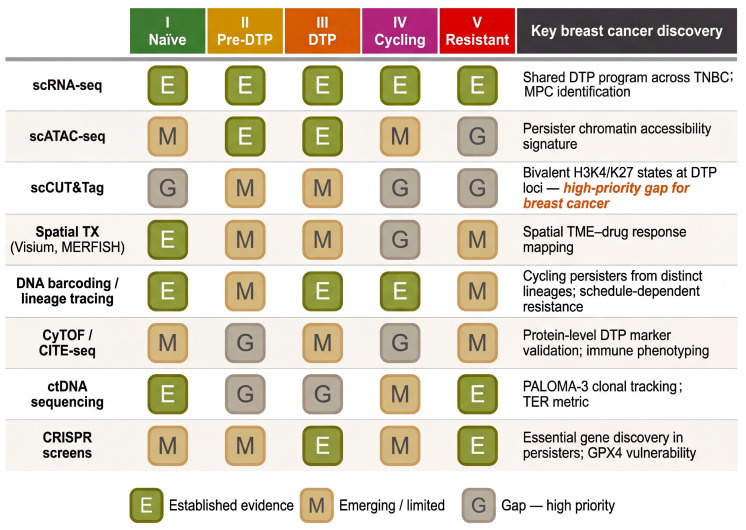
Technology roadmap for dissecting the Resistance Continuum in breast cancer. Matrix mapping the applicability of key single-cell and molecular profiling technologies across the five stages of the Resistance Continuum. Evidence levels are color-coded as stronger support, emerging support, or a major evidence gap. Notable gaps include the limited application of scCUT&Tag across most stages, the inability of ctDNA to directly detect non-genetic DTP states during the early continuum, and the paucity of spatial and single-cell data at the cycling persister stage. Column header colors correspond to the Resistance Continuum stages defined in Figure 1. scRNA-seq: Shared DTP program across TNBC (Baudre et al. 2025 [22]); MPC identification (Wang et al. 2023 [73]). scATAC-seq: Persister chromatin accessibility signature (Grosselin et al. 2019, Rosano et al. 2024 [66,113]). Spatial Tx: Spatial TME-drug response mapping (Jiménez-Santos 2024 [154]). DNA barcoding: Cycling persisters from distinct lineages (Oren et al. 2021 [46]); schedule-dependent resistance (Patwardhan et al. 2021 [163]). ctDNA sequencing: PALOMA-3 clonal tracking (O’Leary et al. 2018 [14]); TER metric (Lv 2025 [50]). CRISPR screens: GPX4 vulnerability (Hangauer et al. 2017 [42]).

The Resistance Continuum Model posits that drug resistance unfolds through five distinct cellular states, each governed by different molecular programs. Translating this conceptual framework into experimentally testable hypotheses requires technologies capable of capturing the genomic, epigenomic, transcriptomic, and spatial dimensions of the resistance trajectory at single-cell resolution. In this section, we map the state-of-the-art multi-omics toolkit onto each stage of the continuum, identify gaps in current technological coverage, and highlight emerging platforms of promise for breast cancer resistance research (Figure 6). Importantly, the technology map underscores that current clinical monitoring tools are asymmetrically informative across the continuum: ctDNA is comparatively powerful for tracking clonal and mutational evolution at later stages, whereas direct detection of pre-DTP and DTP states remains a major unmet need in breast cancer.

### 8.1. Single-Cell RNA Sequencing: Trajectory Inference and Pseudotime Analysis

Single-cell RNA sequencing (scRNA-seq) remains the most widely deployed technology for dissecting tumor heterogeneity and has been instrumental in defining DTP transcriptional signatures in breast cancer. Key applications include the identification of rare pre-DTP subpopulations within treatment-naïve tumors [102,156], the characterization of shared persister programs across TNBC patients and treatment modalities [22], and the resolution of cycling persister emergence through pseudotime trajectory analysis [85]. Serial scRNA-seq profiling of matched pre- and post-treatment breast cancer specimens has further enabled tracking of transcriptional state transitions along the resistance trajectory, revealing that metastatic precursor cells (MPCs) emerge within the post-treatment primary tumor, with gene expression signatures convergent with those of distant metastases [157]. However, scRNA-seq captures only a transcriptomic snapshot and cannot directly resolve the epigenetic or genetic underpinnings of the states it identifies, motivating the complementary technologies described below.

### 8.2. Single-Cell Epigenomics: scATAC-Seq and scCUT&Tag

Given the central role of chromatin remodeling in the DTP state, single-cell epigenomic profiling is essential for dissecting the resistance continuum at its mechanistic core. Single-cell ATAC-seq (scATAC-seq) maps genome-wide chromatin accessibility at single-cell resolution, enabling the identification of cell-type-specific and state-specific regulatory elements [158]. In breast cancer, scATAC-seq has revealed that persister cells exhibit a distinctive chromatin accessibility signature characterized by opening at stress-response enhancers and closing at proliferative transcription factor binding sites [66,113]. The more recently developed scCUT&Tag technology enables profiling of specific histone modifications (e.g., H3K4me3, H3K27me3) at single-cell resolution, offering a direct readout of bivalent chromatin states and KDM5/EZH2-mediated epigenetic rewiring, central to Section 3 and Section 5 [159]. Application of scCUT&Tag to longitudinal breast cancer specimens—before, during, and after therapy—represents a high-priority experimental approach for mapping the epigenetic trajectory of the Resistance Continuum in vivo.

### 8.3. Spatial Transcriptomics and Proteomics: Mapping the Persister Niche In Situ

A fundamental limitation of dissociation-based single-cell methods is the loss of spatial context. Spatial transcriptomics (ST) technologies, including sequencing-based platforms (10× Visium, Slide-seq) and imaging-based methods (MERFISH, seqFISH+), preserve tissue architecture while providing gene expression data, enabling the study of the persister niche described in Section 7 [33,160]. Recent ST applications in breast cancer have mapped subtype-specific spatial heterogeneity [154], identified spatially compartmentalized immune–tumor interactions [146], and resolved the spatial organization of drug-responsive and drug-resistant tumor subpopulations within the same lesion [161]. The integration of spatial transcriptomics with spatial proteomics (e.g., CODEX, IMC) and spatial metabolomics promises to provide a comprehensive, multi-modal spatial map of the resistance continuum—a vision that is technically feasible but not yet realized in breast cancer.

### 8.4. DNA Barcoding and Lineage Tracing

While scRNA-seq and spatial omics provide snapshots of cellular states, DNA barcoding and lineage tracing enable the reconstruction of clonal histories and relationships of fate over time. The landmark study by Oren et al. used expressed barcodes coupled with scRNA-seq to demonstrate that cycling persisters arise from distinct lineages with pre-determined transcriptional programs [46]. Single-cell lineage tracing in cancer xenografts has further revealed that metastatic dissemination rates and routes are lineage-dependent [162]. In breast cancer, DNA barcoding has been applied to track clonal dynamics under different treatment schedules in TNBC cell lines, revealing that the pattern and speed of resistance emergence are schedule-dependent [163]. Expanding these approaches to patient-derived models and, ultimately, to clinical specimens through natural barcodes (e.g., somatic mutations, mitochondrial DNA variants) represents a critical next step for validating the Resistance Continuum in clinically relevant contexts.

### 8.5. Computational Integration and Current Limitations

The power of multi-omics approaches is fully realized only through computational integration. Methods for joint analysis of scRNA-seq and scATAC-seq data (e.g., WNN, MOFA+), spatial deconvolution algorithms (SPOTlight, Cell2location), and clonal inference frameworks (PyClone, CITUP, Startle) are enabling researchers to link transcriptomic states with epigenetic configurations and clonal identities [50,164]. However, significant limitations remain: most single-cell multi-omics datasets are cross-sectional rather than longitudinal; integrating spatial and dissociation-based data remains algorithmically challenging; and the cost and throughput of multimodal profiling limit sample sizes. Overcoming these barriers will be essential for translating the Resistance Continuum from a conceptual model into a quantitatively parameterized, patient-specific framework.

## 9. Therapeutic Strategies Targeting the Window of Vulnerability

**Figure 7:**
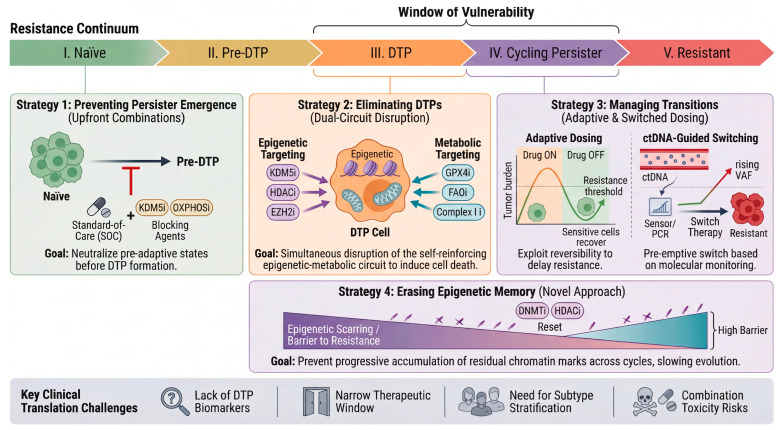
Candidate therapeutic intervention points along the Resistance Continuum.

The Resistance Continuum suggests that there may be an interval between the emergence of non-genetic persistence and the dominance of stable resistant clones during which resistance trajectories remain more modifiable than at later stages. We refer to this interval as a putative “window of vulnerability,” while acknowledging that its timing, duration, and even existence are likely to vary across breast cancer subtypes, treatment modalities, and individual patients. In this section, we discuss candidate therapeutic strategies that may be relevant at different points along this interval, organized by their stage of intervention on the Resistance Continuum (Figure 7).

The schematic maps four classes of intervention onto the proposed Resistance Continuum, with emphasis on the interval between reversible persistence and stable resistance. These strategies should be interpreted as stage-informed therapeutic hypotheses rather than uniformly validated clinical options. Strategy 1 (green) represents prevention of persister emergence through upfront combinations designed to suppress pre-adaptive entry programs. Strategy 2 (purple/teal/amber) represents the elimination of established drug-tolerant persister (DTP) cells through epigenetic, metabolic, or dual-circuit targeting, largely based on preclinical evidence. Strategy 3 (pink) represents efforts to delay or disrupt the transition toward resistant clonal dominance through adaptive scheduling and mutation-informed treatment adjustment, for which ctDNA is most relevant as a monitor of clonal evolution rather than a direct readout of DTP biology. Strategy 4 (red) represents epigenetic memory modulation, a forward-looking conceptual strategy aimed at reducing the long-term persistence-favoring effects of prior drug exposure; this approach remains speculative and requires substantial mechanistic and translational validation. The lower panel summarizes major barriers to clinical translation, including biomarker scarcity, subtype heterogeneity, uncertain timing of intervention, and limited tools for monitoring non-genetic persistence in patients.

### 9.1. Preventing Persister Emergence: Upfront Combination Strategies

The most upstream intervention point is the prevention of DTP emergence itself. The rationale is straightforward: if pre-adaptive states can be identified and neutralized at the time of initial treatment, the persister pool that seeds the entire resistance trajectory may be eliminated before it forms. Preclinical evidence supports the concept of upfront combination therapy pairing standard-of-care agents with drugs targeting molecular programs that enable DTP entry. For example, combining endocrine therapy with KDM5 inhibitors in ER+ breast cancer models has been shown to reduce transcriptomic heterogeneity and diminish the persister fraction [65]. Similarly, co-administration of OXPHOS inhibitors (e.g., IACS-010759) alongside chemotherapy has demonstrated efficacy in reducing DTP survival in preclinical models by disrupting the glycolysis-to-OXPHOS metabolic switch that sustains the persister state [42,165]. Evolutionary principles have also been exploited to prolong tumor control in preclinical breast cancer models through adaptive scheduling [166]. However, these upfront combination strategies face significant translational challenges, including increased toxicity, the difficulty of identifying which patients will benefit, and the lack of biomarkers to confirm DTP prevention in real time.

### 9.2. Eliminating DTP Cells: Targeting Epigenetic and Metabolic Vulnerabilities

Once DTP cells have formed, their unique epigenetic and metabolic dependencies create actionable therapeutic vulnerabilities. Three classes of agents have shown promise in preclinical settings.

First, epigenetic inhibitors targeting the chromatin-modifying enzymes that sustain the DTP state. KDM5 inhibitors disrupt the redistribution of H3K4me3, thereby uncoupling persister cells from lineage-identity programs [21,65]. HDAC inhibitors, by promoting chromatin opening and re-expression of pro-apoptotic and differentiation genes, have been shown to force persister cells out of quiescence and into a drug-sensitive state in multiple cancer types [20,21,110]. However, the relevance of EZH2 inhibitors in TNBC is likely to be highly context- and timing-dependent, because H3K27me3 is a central determinant of early chemotolerance and depletion of H3K27me3 at treatment onset increased chemotherapy tolerance in preclinical models [23]. Accordingly, EZH2 inhibition may be better positioned within rational combination strategies rather than as a simple monotherapy approach in this setting [112].

Second, metabolic disruptors that exploit the OXPHOS and lipid metabolism dependencies of DTP cells. The landmark finding by Hangauer et al. that DTP cells are selectively vulnerable to GPX4 inhibition—and thus to ferroptotic cell death—has catalyzed interest in ferroptosis-inducing agents as persister-targeting drugs [42,107,108]. Inhibitors of fatty acid oxidation (FAO) and mitochondrial complex I represent complementary metabolic targeting strategies, although clinical-grade inhibitors with acceptable toxicity profiles remain limited [167]. The therapeutic implications of metabolic targeting depend on whether a given persister population is dominated by metabolic dependency or by metabolic adaptability. OXPHOS inhibition may be effective in dependency-dominant settings, whereas highly adaptable DTP populations may require combination or sequential metabolic strategies to prevent compensatory rewiring.

Third, agents that disrupt the self-reinforcing epigenetic–metabolic circuit described in Section 5.3. Because the DTP state is sustained by a bidirectional loop between metabolic rewiring and chromatin remodeling, simultaneous perturbation of both arms may be required for durable persister elimination—a principle that argues for rational combination of epigenetic and metabolic agents rather than monotherapy with either class alone.

### 9.3. Preventing the DTP-to-Resistant Clone Transition: Adaptive Dosing Strategies

A conceptually distinct approach targets not the DTP cells themselves but the transition from persister to resistant clone. Adaptive therapy and intermittent dosing strategies, inspired by evolutionary game theory and ecological competition models, exploit the fitness cost of resistance by alternating between periods of drug pressure and drug holidays [168,169]. The rationale is that during drug holidays, drug-sensitive cells that have reverted from the DTP state can outcompete cycling persisters and nascent resistant clones, thereby maintaining a reservoir of sensitive cells that suppresses resistance outgrowth [170,171].

Preclinical data in breast cancer models support this concept. Patwardhan et al. demonstrated that treatment scheduling—including drug holiday length and sequential versus concurrent administration—significantly influenced the pattern and speed of resistance emergence in TNBC cells, with specific schedules delaying resistance by maintaining greater clonal diversity [163]. Enriquez-Navas et al. similarly showed that exploiting evolutionary principles prolonged tumor control in preclinical breast cancer models [166]. Clinical translation of adaptive dosing remains in early stages, but the Resistance Continuum framework provides a mechanistic rationale for its design: drug holidays should be timed to allow DTP cells to revert to a sensitive state (exploiting the reversibility of the DTP phenotype), while treatment re-initiation should occur before cycling persisters have accumulated sufficient genetic diversity to generate stably resistant clones (exploiting the window of vulnerability). For adaptive or intermittent dosing approaches to become clinically actionable within the Resistance Continuum framework, future studies will need to define operational parameters such as trigger biomarkers, ctDNA VAF thresholds, drug-holiday duration, and stopping rules for re-initiation. Without such operational anchors, adaptive therapy remains conceptually attractive but difficult to standardize prospectively in breast cancer.

### 9.4. Exploiting Epigenetic Memory Therapeutically

The concept of epigenetic memory raises the possibility that resistance prevention might eventually extend beyond eliminating persister cells to modulating the long-term chromatin consequences of prior drug exposure. At present, however, this idea should be regarded as a forward-looking experimental hypothesis rather than a clinically established therapeutic strategy. Early-line combination of standard therapy with epigenetic modifiers—administered not to kill persister cells directly but to prevent the accumulation of chromatin scars—represents a conceptually novel approach that has not yet been tested in clinical trials. Candidate agents include DNA methylation inhibitors (decitabine, azacitidine), which could prevent the consolidation of resistance-associated methylation patterns, and histone deacetylase inhibitors, which maintain chromatin in an open, “memory-free” state. This strategy aligns with the growing clinical interest in epigenetic priming and warrants dedicated investigation in breast cancer-specific preclinical models.

However, the therapeutic application of global epigenetic modifiers for memory erasure carries substantial risks that must be carefully weighed. First, HDAC inhibitors, while capable of promoting chromatin opening and re-expression of silenced genes, can paradoxically induce cell cycle arrest and quiescence in certain cellular contexts—the very features that define the DTP state [172,173]. Thus, HDAC inhibition intended to erase chromatin scars could, under some circumstances, reinforce rather than disrupt the persister phenotype. Second, global DNA demethylation through DNMT inhibitors risks reactivation of oncogenes, transposable elements (particularly LINE-1 and Alu elements), and endogenous retroviruses, which could introduce genomic instability and potentially accelerate, rather than prevent, the emergence of resistant clones [174,175]. Third, systemic epigenetic modifiers inevitably affect normal tissue epigenomes, raising concerns about off-target toxicity, particularly in tissues with high turnover rates such as the hematopoietic system and gastrointestinal epithelium. These risks collectively suggest that the clinical application of epigenetic memory erasure will require careful dose optimization, patient selection, and combination strategies that minimize off-target effects.

A further complication arises from the timing-dependent effects of epigenetic intervention. Marsolier et al. demonstrated that depletion of H3K27me3 at the onset of chemotherapy treatment increased rather than decreased chemotolerance in TNBC models, because the removal of this repressive mark derepressed survival and stress-response genes that facilitated initial DTP entry [23]. This finding creates a “timing paradox” for epigenetic memory erasure: global epigenetic modification before or during the first treatment cycle may inadvertently lower the barrier to initial DTP formation by removing repressive marks at survival loci, while the same intervention applied after the first DTP cycle—when the goal is to erase residual scars accumulated during persister-state traversal—could be beneficial. This paradox implies that epigenetic memory erasure strategies must be deployed within a defined temporal window: specifically, during the drug-free interval between treatment cycles, after DTP cells have reverted to an apparently sensitive state but before the next round of drug exposure. Identifying and validating this window in preclinical models is a prerequisite for clinical translation.

Given the limitations and risks of global epigenetic modifiers, locus-specific epigenetic editing represents a conceptually superior alternative for therapeutic memory erasure. CRISPR-based epigenome editing tools—including dCas9 fused to DNMT3A (for targeted DNA methylation), p300 (for targeted histone acetylation), or KRAB (for targeted H3K9me3 deposition and gene silencing)—enable the modification of chromatin state at individual genomic loci without affecting the broader epigenome [176,177]. In principle, these tools could be directed to the specific memory loci identified through the serial drug challenge experiments proposed in Section 5.4, allowing precise erasure of residual scars at stress-response and survival gene promoters while leaving the remainder of the chromatin landscape intact. Accordingly, therapeutic “memory erasure” is best framed as a research agenda for future breast cancer studies rather than an intervention ready for clinical implementation. Priority questions include whether persistence-associated chromatin states are causally linked to re-resistance, whether they can be selectively identified in patient-derived models, and whether partial resetting can be achieved without compromising lineage identity or increasing phenotypic plasticity elsewhere in the tumor ecosystem.

### 9.5. Preclinical Promise and Clinical Translation Challenges

Despite the compelling preclinical rationale for persister-targeting strategies, several challenges impede clinical translation [56]. The transient and rare nature of DTP cells makes pharmacodynamic monitoring difficult; there are currently no validated clinical biomarkers for the DTP state. The therapeutic window between DTP emergence and stable resistance is narrow and patient-specific, requiring real-time monitoring tools such as ctDNA dynamics (Section 6.5) to guide intervention timing. Furthermore, the heterogeneity of DTP mechanisms across breast cancer subtypes (Section 4) implies that a one-size-fits-all approach is unlikely to succeed; subtype-stratified clinical trial designs will be necessary. Addressing these challenges will require close integration of the multi-omics technologies described in Section 8 with innovative clinical trial frameworks—a convergence that represents the next frontier of resistance-informed precision oncology in breast cancer.

## 10. Conclusions and Future Perspectives

### 10.1. Summary of the Resistance Continuum Model

In this review, we have outlined the Resistance Continuum as a breast cancer-focused framework for organizing evidence across treatment-naïve heterogeneity, pre-adaptive priming, reversible drug-tolerant persister (DTP) states, cycling persisters, and genetically stabilized resistant clones. This model traces the complete evolutionary trajectory from treatment-naïve heterogeneity [37,40], through pre-adaptive epigenetic and metabolic priming [23,65], into the reversible drug-tolerant persister (DTP) state [15,21], followed by cycling persister expansion [46], and culminating in the emergence of genetically fixed resistant clones [30,50]. By integrating evidence from single-cell and spatial multi-omics [34,160], clonal dynamics studies [62,127], and preclinical therapeutic models, we have demonstrated that this trajectory is not a theoretical abstraction but a biologically defined process with measurable molecular features at each stage. Critically, the model identifies a “window of vulnerability”—the interval between DTP formation and the onset of stable genetic resistance—as the optimal target for therapeutic intervention. We have further shown that this trajectory manifests with both shared and subtype-specific features across ER+, HER2+, and TNBC breast cancers, necessitating a subtype-informed approach to anti-resistance strategies.

### 10.2. Key Unanswered Questions

Despite the substantial progress reviewed herein, several critical questions remain unresolved, each representing a high-priority direction for future investigation:

(i) What is the relative contribution of pre-existing genetic variants versus therapy-induced mutagenesis (e.g., APOBEC3A) to the emergence of resistant clones in each breast cancer subtype, and can these contributions be quantified using longitudinal single-cell multi-omics?
(ii) Can the DTP state be reliably detected in clinical specimens using minimally invasive assays (e.g., ctDNA epigenetic signatures, circulating tumor cell profiling), and can such detection be used to guide real-time therapeutic decision-making?
(iii) To what extent does “epigenetic memory” from prior DTP episodes accelerate resistance to subsequent lines of therapy, and can this memory be therapeutically erased without unacceptable toxicity?
(iv) How does the spatial organization of the tumor microenvironment—particularly the CAF, immune, and hypoxic niche architecture—influence the probability and kinetics of DTP emergence across breast cancer subtypes?
(v) Can adaptive dosing strategies, designed based on evolutionary game theory and informed by real-time ctDNA monitoring, outperform continuous dosing regimens in clinical trials for metastatic breast cancer?

### 10.3. Vision: From Reactive Treatment to Proactive Resistance Prevention

The current clinical paradigm for managing drug resistance in breast cancer remains largely reactive: therapy is typically changed after resistant disease has become clinically apparent. The framework developed here suggests a complementary perspective: in some settings, resistance may be more effectively addressed by identifying and intercepting earlier adaptive states before stable clonal dominance is established. Realizing this goal will require more than conceptual reframing. It will depend on longitudinal patient-linked datasets, improved biomarkers for non-genetic persistence, clearer subtype-specific models, and careful integration of mechanistic and clinical evidence.

We emphasize, however, that these enabling capabilities vary in maturity. ctDNA-based monitoring of clonal and mutational evolution is already clinically actionable in selected settings, whereas persister-targeting strategies remain largely preclinical or early translational, and individualized trajectory-prediction frameworks are still aspirational. We therefore view the Resistance Continuum not as a finalized map of breast cancer resistance, but as a scaffold for future testing that may help guide a gradual shift from retrospective description of resistance to earlier, evidence-based intervention. This framework is intended to organize current evidence and identify testable translational priorities, rather than to define a uniform or immediately deployable clinical roadmap.

## Figures and Tables

**Table 1:** Comparison of the Resistance Continuum Model with prior resistance frameworks.

Feature	Marine et al. (2020) [18]	Vasan et al. (2019) [7]	Pu et al. (2023) [20]	Laplane & Maley (2024) [17]	This Review
Scope	Pan-cancer	Pan-cancer	Pan-cancer	Pan-cancer	Breast cancer-specific
Formally defined stages with temporal dynamics	◑ Conceptual phases, no staging	◑ Mechanism categories, not temporal	◯ DTP characterization, no staging	◑ Two-phase (tolerance → resistance)	● Five stages (I–V) with hours-to-months timescales
Epigenetic memory ratchet	◯ Not addressed	◯ Not addressed	◯ Not addressed	◯ Not addressed	● Residual chromatin scars accelerate re-resistance (Section 5.4)
Epigenetic–metabolic circuit integration	◑ Epigenetic and metabolic discussed separately	◑ Metabolic noted as resistance mechanism	◑ Metabolic reprogramming surveyed	◯ Not integrated	● Bidirectional α-KG/SAM/FAD ↔ chromatin circuit (Section 5.3)
Breast cancer subtype stratification	◯ Pan-cancer examples	◑ Some breast cancer examples	◯ Pan-cancer examples	◯ Cancer-general framework	● ER+, HER2+, TNBC compared (Table 1, Section 4)
Technology–stage evidence gap mapping	◯ Not addressed	◯ Not addressed	◯ Not addressed	◯ Not addressed	● 8 technologies × 5 stages matrix (Section 8)
Therapeutic window concept	◑ Implied; no stage-mapped strategies	◑ Resistance prevention discussed broadly	◑ DTP-targeting strategies reviewed	◑ Persistence → resistance trajectory	● Four stage-specific strategies + memory erasure (Section 9)
TME as modulator of resistance trajectory	◑ Briefly mentioned	◑ TME as resistance mechanism	◑ Niche signaling noted	◯ Not addressed	● CAF, immune, hypoxia niche mapped along continuum (Section 7)

Each row evaluates a conceptual feature across four existing frameworks and the present model. Filled circles (●) indicate that the feature is substantively addressed; half-filled circles (◑) indicate partial or implicit coverage; empty circles (◯) indicate that the feature is absent or not addressed. → indicates a directional transition or shift; ↔ indicates bidirectional interaction.

## Data Availability

No new data were created or analyzed in this study. Data sharing is not applicable to this article.

## Abbreviations

The following abbreviations are used in this manuscript:

ADC	Antibody–drug conjugate
AKT	Protein kinase B
AP-1	Activator protein 1
APOBEC3A	Apolipoprotein B mRNA editing enzyme catalytic subunit 3A
CAF	Cancer-associated fibroblast
CDK2	Cyclin-dependent kinase 2
CDK4/6	Cyclin-dependent kinases 4 and 6
CDK4/6i	CDK4/6 inhibitor(s)
CITUP	Clonal inference in tumors using phylogeny
CODEX	CO-Detection by indEXing
CSC	Cancer stem cell
ctDNA	Circulating tumor DNA
DCIS	Ductal carcinoma in situ
DNA	Deoxyribonucleic acid
DNMT	DNA methyltransferase
DTP	Drug-tolerant persister
ECM	Extracellular matrix
EGFR	Epidermal growth factor receptor
EMT	Epithelial-to-mesenchymal transition
ER	Estrogen receptor
ER+	Estrogen receptor-positive
ET	Endocrine therapy
EZH2	Enhancer of zeste homolog 2
FAD	Flavin adenine dinucleotide
FAO	Fatty acid oxidation
FGFR	Fibroblast growth factor receptor
GPX4	Glutathione peroxidase 4
H3K4me3	Histone H3 lysine 4 trimethylation
H3K27me3	Histone H3 lysine 27 trimethylation
H4K20me3	Histone H4 lysine 20 trimethylation
HDAC	Histone deacetylase
HER2	Human epidermal growth factor receptor 2
HER2+	HER2-positive
HGF	Hepatocyte growth factor
HIF-1α	Hypoxia-inducible factor 1 alpha
HLA-G	Human leukocyte antigen G
HMOX1	Heme oxygenase 1
ICI	Immune checkpoint inhibitor
IGF-1R	Insulin-like growth factor 1 receptor
IL-6	Interleukin 6
IL-10	Interleukin 10
IMC	Imaging mass cytometry
ISR	Integrated stress response
ITH	Intra-tumoral heterogeneity
JAK	Janus kinase
KDM5A/B	Lysine demethylase 5A/B (JARID1A/B)
KDM6A/B	Lysine demethylase 6A/B
KRT5/14/17	Keratin 5/14/17
LAM	Lipid-associated macrophage
LSD1	Lysine-specific demethylase 1 (KDM1A)
MAPK	Mitogen-activated protein kinase
MDR1	Multidrug resistance protein 1 (P-glycoprotein)
MERFISH	Multiplexed error-robust fluorescence in situ hybridization
MET	Mesenchymal–epithelial transition factor (hepatocyte growth factor receptor)
MHC	Major histocompatibility complex
MOFA+	Multi-Omics Factor Analysis
MPC	Metastatic precursor cell
mTOR	Mechanistic target of rapamycin
NF-κB	Nuclear factor kappa-light-chain-enhancer of activated B cells
NRF2	Nuclear factor erythroid 2-related factor 2 (NFE2L2)
OXPHOS	Oxidative phosphorylation
PD-1	Programmed cell death protein 1
PD-L1	Programmed death-ligand 1
PDX	Patient-derived xenograft
P-gp	P-glycoprotein (MDR1/ABCB1)
PI3K	Phosphatidylinositol 3-kinase
PINK1	PTEN-induced kinase 1
PIK3CA	Phosphatidylinositol-4,5-bisphosphate 3-kinase catalytic subunit alpha
PRC2	Polycomb repressive complex 2
RB1	Retinoblastoma protein 1
RNA	Ribonucleic acid
ROS	Reactive oxygen species
RTK	Receptor tyrosine kinase
SAM	S-adenosylmethionine
scATAC-seq	Single-cell assay for transposase-accessible chromatin sequencing
scCUT&Tag	Single-cell cleavage under targets and tagmentation
scRNA-seq	Single-cell RNA sequencing
SLC7A11	Solute carrier family 7 member 11 (xCT)
ST	Spatial transcriptomics
STAT3	Signal transducer and activator of transcription 3
TAM	Tumor-associated macrophage
TCA	Tricarboxylic acid
T-DXd	Trastuzumab deruxtecan
TER	Tumor clonal evolution rate
TGF-β	Transforming growth factor beta
TIGIT	T-cell immunoreceptor with Ig and ITIM domains
TIM-3	T-cell immunoglobulin and mucin domain-containing protein 3
TME	Tumor microenvironment
TNBC	Triple-negative breast cancer
VAF	Variant allele frequency
WNN	Weighted nearest neighbor
xCT	Cystine/glutamate antiporter (SLC7A11)
α-KG	Alpha-ketoglutarate
